# The Influence of Spatial Distance and Environment on Small‐Scale Genetic Variability in Eelgrass and Its Application for Restoration

**DOI:** 10.1111/eva.70127

**Published:** 2025-07-11

**Authors:** Marlene Jahnke, Stefanie R. Ries, Swantje Enge, Christian Pansch, Giannina Hattich, Maru Bernal‐Gómez, Pierre De Wit, Jonathan Havenhand

**Affiliations:** ^1^ Department of Marine Sciences—Tjärnö Marine Laboratory University of Gothenburg Strömstad Sweden; ^2^ Environmental and Marine Biology Åbo Akademi University Åbo/Turku Finland; ^3^ Department of Biological & Environmental Sciences University of Gothenburg Gothenburg Sweden

**Keywords:** common garden experiment, donor site selection, EU Nature Restoration Law, local adaptation, preadaptation, *Zostera marina*

## Abstract

Identifying suitable donor sites is an important component of successful restoration and reduces the likelihood that a restoration action will have negative impacts on surrounding populations. Whether the most suitable donor site has (1) fast‐growing phenotypes, (2) high genetic diversity, or (3) harbors alleles that are beneficial for the current or future environment at the restoration site is an ongoing debate in restoration genomics. It is also debated whether one single donor site is the best choice, or if a mixed provenance strategy from sites with different characteristics is preferable. For eelgrass restoration, donor material is typically sourced within a few kilometers. It is therefore also this small spatial scale that needs to be considered when testing which local meadows harbor the most beneficial donor material for a given restoration site. We here assessed micro‐habitat differences at 10 eelgrass meadows across 1.5–14 km and genotyped the 10 meadows at 1689 single nucleotide polymorphisms (SNPs). We observed substantial differences in temperature regimes, genetic differentiation, and genetic diversity. We found that even on this small scale, 10% of the overall genetic variation was explained by the local environment of the meadow as well as geographic distance and genetic differentiation. We also identified putative adaptive loci associated with environmental variables and detected differences in growth in common‐garden mesocosm experiments simulating ambient summer conditions as well as a marine heatwave with concurrent freshening. We highlight that the variation in environment, genetic diversity, local adaptation, the potential for preadaptation for future conditions, and differences in individual growth can be strong in eelgrass meadows even on the small spatial scale. We suggest a donor registry to take into account these differences and narrow down the pool of potential donor meadows to source the most beneficial combination of donor material for any given restoration site.

## 1 Introduction

Marine conservation and seascape genomic assessments are generally carried out at large spatial scales (100s km; Jahnke and Jonsson 2022). These studies provide valuable insights into connectivity patterns, broad population structure across wide geographic regions, and adaptation across large‐scale environmental gradients (Liggins et al. 2019; Nielsen, Hanson, et al. 2023). However, these large‐scale assessments often do not match the level at which management can make use of conservation genomics, for instance, in the establishment of marine protected areas and restoration actions, which are generally regional or local. Regional‐scale genetic assessments (10s–100s km) are valuable for spatial‐based conservation planning (Combrink et al. 2024; Faust et al. 2025; Nielsen, Beger, et al. 2023); however, they may still not provide the resolution necessary for localized management actions, particularly in the context of restoration. Sourcing donor material for restoration currently often occurs at much smaller spatial scales—typically within a few kilometers (Breed et al. 2019; Rossetto et al. 2019). Therefore, this scale will need to be increasingly considered in restoration genomics (Breed et al. 2019; Wood et al. 2020) in order to identify which nearby site (s) harbor genetic material that is most likely to be beneficial at a restoration site. Identifying the most suitable donor sites for restoration is an important component for increasing the likelihood that a restoration action is successful and has minimal negative impact on remaining surrounding populations (Wood et al. 2020).

**FIGURE 1:**
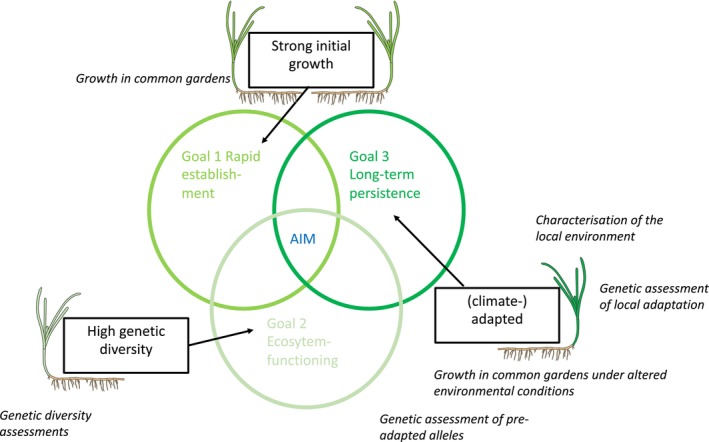
Conceptual figure illustrating the three main goals of restoration. In black boxes are the respective three desirable donor material characteristics targeting each of the three specific restoration goals. We explore if a single donor site or which combination of donor material is likely to achieve all three goals, based on growth in common garden experiments, genetic diversity assessments, characterization of the local environment, genetic adaptation, and the identification of preadapted alleles (in italics). The figure was inspired by Kettenring et al. (2014).

When carrying out restoration actions, practitioners directly or implicitly aim to achieve three main goals: (1) maximize initial plant growth and establishment, (2) restore ecosystem functioning, and (3) ensure long‐term persistence (Kettenring et al. 2014; Figure 1). From a large body of research, the appropriate donor material for each aim has been inferred in general terms (Kettenring et al. 2014). Selecting individuals with strong growth and expansion is likely to lead to rapid establishment (Agneray et al. 2022; Kettenring et al. 2014). High genetic diversity has been shown to increase ecosystem functioning, for instance, measured as species diversity, primary productivity, or the resistance and resilience to disturbance (Hughes et al. 2008; Hughes and Stachowicz 2004; Reusch et al. 2005; Reynolds, McGlathery, et al. 2012). Finally, proactively matching donor material with the local environment, and particularly forecasted future environmental conditions, is acknowledged as the only way to ensure the third goal of long‐term persistence of restored habitats (Coleman et al. 2020; Pazzaglia et al. 2021; van Oppen et al. 2017). While the goals overlap to a certain extent (Kettenring et al. 2014; Figure 1), it remains mostly unexplored as to whether the three main goals are also to a degree mutually exclusive, and whether therefore the most suitable donor site (1) harbors fast‐growing phenotypes, (2) has high genetic diversity, (3) harbors alleles that are conducive for the current or future environment at the restoration site, or (4) consists of mixed provenance sourcing from multiple donor sites with different characteristics (Bailey et al. 2021; Kettenring et al. 2014).

Eelgrass ( *Zostera marina* ), a marine flowering plant found in coastal waters of the Northern hemisphere, plays a critical role in coastal ecosystems by providing habitat for a range of species and by contributing to nutrient cycling, sediment stabilization, and carbon sequestration (Meysick et al. 2022; Moksnes et al. 2021; Unsworth et al. 2012). In Scandinavia, eelgrass generally has a fragmented and patchy distribution due to habitat limitations, the dynamic nature of coastal environments, and human impact (Boström et al. 2014). Fragmentation and loss due to physical impact, pollution, and climate change have been documented on different time scales (Nordlund et al. 2024). A dramatic loss of eelgrass driven by wasting disease already occurred in the 1930s (Rasmussen 1977). Eelgrass recovered to some extent in Scandinavia in the 1960s–1980s, but never regained its historic distribution (Boström et al. 2014). More recent declines have occurred over the last decades, and along the Swedish northwest coast approximately half of eelgrass has vanished since the 1980s (Baden et al. 2003; Moksnes and Bergström 2025). Eutrophication, overfishing, and the accumulation of many small‐scale developments, such as piers and jetties, have been identified as the main drivers of decline (Baden et al. 2010; Eriander et al. 2017; Eriksson et al. 2023; Moksnes et al. 2008). Future predicted drivers for eelgrass loss are direct or indirect effects of decreasing salinity and increasing temperature caused by climate change (Jeffery et al. 2023; Torn et al. 2020). To mitigate ongoing and predicted eelgrass losses, a number of measures are presently being discussed and implemented, including protected‐area based conservation and large‐scale restoration (Faust et al. 2025; Moksnes et al. 2016; Ries et al. 2023). While seagrass restoration is being carried out across wide geographic scales (Nordlund et al. 2024; van Katwijk et al. 2021), its success to date is mixed (van Katwijk et al. 2016). Given global change, restoration focused on boosting resilience to future climate by sourcing donor material “preadapted” to future conditions is being increasingly explored (Nimbs et al. 2024; Pazzaglia et al. 2021).

While eelgrass meadows are a relatively common habitat across the Northern hemisphere, there is large variation in eelgrass growth form, ranging from an average shoot length of 10 to 247 cm and an average below‐ground biomass from 8 to 1122 g per plant across different meadows in the Northern hemisphere (Duffy et al. 2022). Importantly, genetic differentiation was identified as the main driver of variation in eelgrass growth form on this large spatial scale (Duffy et al. 2022). Eelgrass meadows also vary strongly in appearance on the small spatial scale of kilometers. For instance, Sotka et al. (2024) report a difference in shoot length of *ca*. 70% among sites separated by < 15 km. Reciprocal transplants on a similar spatial scale of 2–12 km indicated also a strong home advantage in growth and survival (DuBois et al. 2022). Notably, also on these small spatial scales, morphological traits seem to have a genetic basis (Abbott and Stachowicz 2016; Hughes and Stachowicz 2009), and phenotypic differences could therefore be the result of small‐scale micro‐habitat adaptation (Hays et al. 2021), despite the possibility of dispersal (Sotka et al. 2024). Indeed, the coastal habitat of eelgrass along the shoreline is shaped by fine‐scale characteristics and therefore consists of mosaics of distinct micro‐habitats including thermal microclimates (DuBois et al. 2022; Hattich et al. 2025). These micro‐habitats likely show variation on the scales of meters and kilometers in environmental variables, such as substrate type, nutrient availability, temperature, salinity, depth, light availability, and current regime, that have been linked to variations in eelgrass phenotype and meadow appearance (Boyé et al. 2022). However, the resolution of environmental data available from monitoring efforts is rarely at the appropriate scale to detect and record variation at the micro‐habitat scale (Dauphin et al. 2023). Moreover, the effects of small‐scale *temporal* variability have often been overlooked, and yet the magnitude of diurnal—or seasonal—variation often exceeds projected climate trends for the coming century (Boyd et al. 2016, 2018; Jahnke et al. 2024). Hence, in situ environmental monitoring at the local scale is vital if we are to address the question of how micro‐habitats are linked to genotype, adaptation, and ultimately fitness. As small‐scale thermal habitat variability can lead to differential sensitivity to global change (DuBois et al. 2022), fine‐scale environmental and genomic assessments are urgently needed to provide information for restoration actions in light of global change.

In Europe, the EU Nature Restoration Law was adopted by the European Parliament and the Council of the European Union in 2024 (EU 2024). This legal framework aims to restore at least 20% of sea areas by 2030, and all ecosystems in need of restoration by 2050 (EU 2024). As a first step, the Nature Restoration Law requires the development of national restoration and monitoring plans by each member state by 2026 (EU 2024). Seagrass is mentioned specifically as a habitat type in Annex II (EU 2024). Given the short time‐lines for implementation, policymakers have been urged to consider that conservation management efforts have the highest probability of success when they are supported by scientific data (van Oppen and Coleman 2022). We here analyze as a case in point eelgrass genomic, phenotypic, and environmental data on the small geographic scale (below 15 km) with the overall aim to identify donor material that matches all three goals of restoration. We use empirical data to identify (1) eelgrass meadows harboring plants with strong growth and (2) eelgrass meadows with high genetic diversity. Identifying donor material for the third goal of long‐term persistence is still state‐of‐the‐art basic research, and we aimed to (3) identify eelgrass meadows that show signs of genetic differentiation appropriate for the presumed genetic background of the chosen restoration site, have a high frequency of putative adaptive alleles, and where growth is not negatively affected by increased temperature and reduced salinity. We explore and discuss how knowledge on natural variability in growth, genetic diversity, and local adaptation among eelgrass meadows can be used for informing the provenance strategy of donor material with the aim to improve restoration practices to achieve all three main goals of restoration (Figure 1).

## 2 Materials and Methods

### 2.1 Sampling of Eelgrass and Assessment of Environmental Variables

**FIGURE 2:**
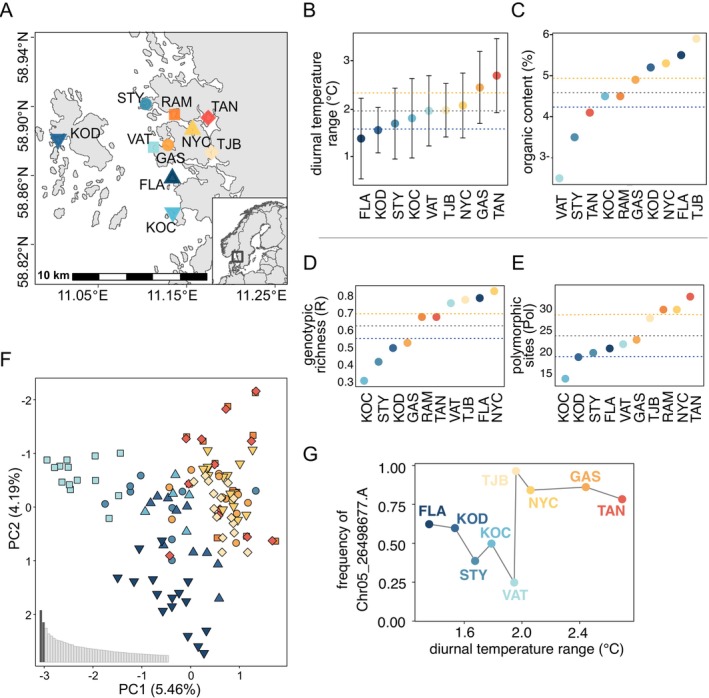
(A) Map of the 10 eelgrass meadows assessed on the small spatial scale along the Swedish North‐West coast. (B, C) Characterization of environmental variables at each meadow during summer 2021. Exposed sites are shown in blues, where increasing darkness indicates sites with decreasing daily temperature range. Sheltered sites are shown in oranges, where increasing darkness indicates increasing daily temperature range (i.e., darkest color is the site with the highest daily temperature range). The gray dotted line indicates the overall mean, whereas the blue dotted line indicates the mean of the exposed sites and the orange dotted line the mean of the sheltered sites. Within each plot, the meadows are ordered according to the value shown on the *y*‐axis. (B) Mean and standard variation of diurnal temperature range and (C) organic content (Table S1). (D, E) Genetic diversity values observed at the 10 eelgrass meadows. The gray dotted line indicates the overall mean, whereas the blue dotted line indicates the mean of the exposed sites and the orange dotted line the mean of the sheltered sites. Within each plot, the meadows are ordered according to the value shown on the *y*‐axis, (D) genotypic richness, and (E) polymorphic loci. (F) Principal component analysis (PCA) of 111 eelgrass multilocus lineages and 124 samples genotyped with 1648 SNPs. Each dot represents one individual and each of the 10 eelgrass meadows is represented by a unique combination of color and symbol (see Figure 2A). (G) Allele frequency of the environment‐associated and putatively adaptive outlier locus Chr05_26498677 (identified by both *pcadapt* and *rdadapt*), which shows a strong association with daily temperature range as identified with *rdadapt*. See Figure S9 for further plots of other identified environment‐associated outlier loci.

The study was performed in the Koster Sea archipelago, assessing eelgrass meadows around the islands of Koster, Tjärnö, Saltö, Öddö, Rossö, and Styrsö on the west coast of Sweden. We collected eelgrass at a depth of 1.5–2.6 m from 10 meadows in July 2021 (Koster) or in May–June 2022 (other sites; Figure 2A, Table S1). The distance between meadows ranged from a minimum of 1.5 km to a maximum of 14 km. At each site, 20 individual shoots of eelgrass were sampled using a “random swim” collecting individual shoots at *ca*. 1.5 m distance in order to reduce the probability of sampling shoots belonging to the same clone (Arnaud‐Haond et al. 2007; Jahnke et al. 2018). The distance of 1.5 m was used for comparative purposes to other assessments, and it is a standardized sampling distance used in the majority of genetic/genomic assessments of eelgrass (e.g., Jahnke et al. 2018; Yu et al. 2023). Environmental data for the characterization of the 10 meadows was taken from (Hattich et al. 2025). In brief, meadows were classified as either sheltered or exposed based on land shelter and openness to the sea (Hattich et al. 2025; Figure 2A). Water temperature measurements were obtained at 15‐min intervals during summer 2021 at all 10 meadows using HOBO MX2201 loggers (ONSET, USA) installed 1 m above the bottom (Hattich et al. 2025). No temperature data were available for 1 out of the 10 meadows, RAM, as the logger became covered by sediment. Winter water temperatures were also collected at the same locations and in the same manner from 2024 to 2025. Due to challenges with winter field work, we were only able to obtain data from 5 of the 10 sites (Table S1). We calculated meadow‐specific daily mean temperatures, daily maximum temperatures, and diurnal temperature ranges for the period 5–29 July 2021 and 20 December 2024–13 February 2025 (Table S1; Hattich et al. 2025). We estimated organic content of the sediment by percentage loss of weight on ignition, that is, percentual weight differences between dried sediment samples (105°C for 22 h) and burned (550°C for 6 h) samples (Table S1).

### 2.2 GOAL 1: Rapid Establishment of Eelgrass

#### 2.2.1 Identification of Eelgrass Meadows With Strong Growth

In order to test for differences in growth among eelgrass meadows at small spatial scales (goal 1), we carried out a common‐garden outdoor mesocosm experiment with shoots collected from the 10 eelgrass meadows. To address long‐term persistence (goal 3) in the same experiment, we also implemented an extreme event treatment in which we assessed growth in ambient summer conditions compared to that in a marine heatwave with concurrent freshening (see below). To identify whether growth is affected positively or negatively by growing together with eelgrass from other meadows, we also applied a treatment of a mixed provenance strategy versus using a single donor meadow (see below). We initially also included a grazer diversity treatment, however, this was dropped due to lack of significance in linear models.

In total, 480 eelgrass shoots were collected for the experiment from the 10 different meadows between 28 June 2021 and 04 July 2021 (Figure 2A; i.e., 48 shoots per meadow). The shoots were acclimatized at Tjärnö Marine Laboratory (58°52′33.6″ N, 11°08′45.6″ E) in constant flow‐through of surface seawater (taken from 1 m depth) until 06 June 2021. Prior to planting, each shoot was weighed, a picture was taken, and a hole was punched with a needle 1 cm above the meristem for later growth measures (Gaeckle and Short 2002). Six shoots were planted into each of 80 cylinders (9.8 L volume, diameter 19 cm, height 34.5 cm), either as a single donor meadow treatment (all 6 shoots from the same meadow) or as mixed provenance treatment (the 6 shoots came from 6 different meadows). Shoots chosen for the mixed provenance treatments were interspersed to ensure even distribution of meadows across replicates. We also added four juveniles of each of two species of grazers, *Idotea balthica* and *I. granulosa* , reared from families from multiple parents collected at two local sites. The 80 different cylinders were distributed over 10 large (900 L) outdoor mesocosm tanks ensuring an even distribution of diversity treatments, and meadow‐origins across the tanks. Each cylinder received separate seawater inflow, with outflow into the mesocosm tanks serving to stabilize water temperature.

To allow acclimatization to the experimental conditions, all cylinders received ambient surface seawater for 10 days. This treatment was maintained for half of the tanks (and the 40 cylinders within them) for the duration of the experiment. The other half of the tanks (40 cylinders) were subjected to a simulated extreme event. Data from Tjärnö Bay have shown that marine heatwaves in the summer (mean +4°C relative to ambient) coincide with local freshening (by −4.7‰; Hattich et al. 2025; Jahnke et al. 2024). Therefore, for the extreme event treatment, inflowing seawater temperature was raised, and the salinity lowered over 2 days to aim for +4°C and −4.7‰ relative to ambient controls. These conditions were then maintained for 9 days during which time temperature was recorded by 23 HOBO MX2201 temperature loggers, randomly distributed across cylinders, and salinity was recorded every second day with a handheld conductivity meter (WTW, Xylem Analytics, Germany). At the end of the experiment, eelgrass shoots were removed, and the growth of each individual was measured as total growth across old and new leaves per shoot.

### 2.3 GOAL 2: Restore Ecosystem Services

#### 2.3.1 Identification of Eelgrass Meadows With High Genetic Diversity—Laboratory Work and Bioinformatics

We reanalyzed genetic data presented in (Hattich et al. 2025) for the assessment of goal 2. To characterize the patterns of genetic diversity of the 10 eelgrass meadows, we carried out DNA extractions and 2b‐RAD library preparations (Wang et al. 2012) as described in Faust et al. (2025). In brief, for each sampling site, 22 DNA samples from 20 individuals were amplified using sample‐specific barcoded adaptors (2 of the 20 sampled individuals were sequenced twice using different barcodes and were used as technical replicates). Single‐read sequencing (51 bp read‐length) was performed on the NovaSeq 6000 system with v1.5 sequencing chemistry (Illumina Inc.). We carried out bioinformatic analyses following the 2b‐RAD GATK pipeline developed by Mikhail Matz (2021) available at https://github.com/z0on/2bRAD_denovo. Briefly, we concatenated reads sequenced on different lanes and removed PCR duplicates (detectable through the use of degenerate primers during library preparation), restriction sites and reads that had more than seven observations, but lacked reverse complement reads. Trimmed reads with a minimum length of 25 bp were then aligned to the *Z. marina* reference genome v.3.1 (Ma et al. 2021) with the local option in *bowtie2* (version 2.5.1; Langmead and Salzberg 2012). As the average depth was above 10 across the data set, we used the GATK hard genotype‐calling pipeline. We generated a first round of putative variants using GATK's *UnifiedGenotyper* (version 3.8‐0), followed by filtering for a minimum depth of 3. We then carried out variant quality score recalibration (VQSR) of the pairs of technical replicates. Some technical replicates were excluded from the training data set due to high missingness and poor heterozygous match (≤ 0.9). *VariantRecalibrator* was used to generate an adaptive error model using the SNPs that were reproducibly genotyped across all technical replicates. Final filtering of the recalibrated data set in *vcftools* (Danecek et al. 2011) was done to select biallelic loci genotyped in at least 90% of individuals, remove individuals with a missingness above 25%, remove SNPs with a coverage higher than twice the mean of the entire data set to remove paralogs, thinning to keep the SNP per RAD locus with highest minor allele frequency (script thinner.pl with criterion = maxAF, https://github.com/z0on/2bRAD_denovo) when relevant, and to remove monomorphic loci.

#### 2.3.2 Identification of Eelgrass Meadows With High Genetic Diversity—Clone Identification and Genetic Diversity Estimates

Since eelgrass can reproduce both sexually and clonally, and ramets can spread across several meters, we estimated the amount of clonal replicates in the data set. We used an approach commonly used in marine macrophytes for clone identification (e.g., Pereyra et al. 2025; Ruocco et al. 2022), which is based on a genetic distance tree using an Unweighted Pair Group Method with Arithmetic Mean (UPGMA) algorithm in the *poppr* package (version 2.9.4; Kamvar et al. 2014) in R v.4.3.0 (R Core Team 2024) and RStudio 2023.3.0.386 (Posit team 2024) to cluster all genotyped individuals based on the Hamming distance (the number of nucleotide differences). We included 20 technical replicates for library preparation and sequencing to then estimate the sequencing error threshold based on the technical replicates, i.e., confirmed clones. The average distance threshold (0.011) was comparable to other studies (Ries et al. 2023; Faust et al. 2025) and was used to define samples as clones of each other. All other samples were considered unique multilocus lineages (MLLs). As is generally done for partially clonal organisms (Meirmans 2024), we then removed replicate clones within a meadow, leaving only one individual of each MLL (the clone‐corrected data set). However, as genetic variability can still arise also during clonal reproduction through somatic mutations (Reusch et al. 2021; Yu et al. 2024), and potentially be adaptive (Nimbs et al. 2024), we also performed all analyses with all successfully sequenced individuals.

Genotypic richness (*R*) on a meadow scale was calculated following Dorken and Eckert (2001), with *R* = (number of MLLs‐1)/(number of samples − 1) for each sampling site. The genetic diversity measures of inbreeding coefficient (*F*_IS_), the percentage of polymorphic sites corrected for sample size (Pol), and rarefaction‐corrected private alleles (Pa) were calculated for both the clone‐corrected 111 MLL data set and all 189 samples with *snpR* (version 1.2.9.2; Hemstrom and Jones 2023).

### 2.4 GOAL 3: Long‐Term Persistence of Restored Eelgrass Meadows

#### 2.4.1 Identification of Locally Adapted Eelgrass Meadows—Genotype–Environment Association Analysis

To investigate genetic differentiation among meadows sampled at this small spatial scale, we performed an analysis of PCA based on all SNPs. For visualization of the PCA, we used the package *adegenet* (version 2.1.10; Jombart and Ahmed 2011), the function *glPca*, and plotted the results with *ggplot2* (version 3.5.1; Wickham 2016). We tested for isolation‐by‐distance using *mantel.test* from the *ncf* package (version 1.3.2; Bjornstad 2022) between geographic distance, measured as sea distance without crossing land in GoogleMaps (Table S2), and pairwise Weir & Cockerham *F*_ST_ calculated with the *hierfstat* package (version 0.5.11; Goudet 2005; Table S3).

To investigate genotype–environment associations, we then used redundancy analysis (RDA), one of the most commonly used multivariate landscape genomic methods to analyze many loci and several environmental variables simultaneously (Capblancq and Forester 2021). We calculated RDAs using the *RDA* function in the R package *vegan* (version 2.6‐4; Oksanen et al. 2024). As response variable, we used an individual‐based data set using genotypes (coded as the count of one allele, i.e., 0/1/2) and considered several predictor variables. For the predictor of geographic distance, we reduced dimensions of the measured sea distance by calculating PCoA distances across all sites using the R package *ape* (version 5.7‐1; Paradis and Schliep 2019; Figure S1). To calculate a “neutral” population structure predictor, we first filtered the full marker set to only maintain intergenic, that is, more likely neutral SNPs, which we located with *locateVariants* from the package *VariantAnnotations* (version 1.52.0; Obenchain et al. 2014). We then performed a PCA of the intergenic SNPs (total of 1110 SNPs for the clone filtered data set and 1135 SNPs for the data set including all 189 samples) with the *RDA* function and selected the first two PC axes, which explained a considerable percentage of genetic differentiation in both data sets. Finally, our local‐scale environmental predictors consisted of the in situ temperature data recorded with HOBO temperature loggers during the summer of 2021 at 9 out of 10 sites. Data for the 10th site was imputed using *mice* (3.16.0; van Buuren and Groothuis‐Oudshoorn 2011). Organic content of the sediment at each meadow was also included as an environmental predictor (Table S1). We then removed variables that had strong correlations above 0.7 (*corrplot* 0.92; Dormann et al. 2013) with other included variables (Figure S2). Collinearity was further checked by removing variables with the largest variance inflation factor (Legendre and De Cáceres 2013), until all variables had values for the square root of the variance inflation factor below two. Variable reduction through tests of collinearity and variance inflation resulted in the retention of two environmental variables (diurnal temperature range and organic content), two PCoAs for sea distance, and two PCs for genetic structure based on intergenic SNPs, which were included in our final full RDA model as predictors of genetic variability.

Next, we carried out variance partitioning with partial RDAs (pRDAs) to identify the contribution of the spatial, environmental, and genetic factors by themselves to genetic variability among populations. Variance partitioning estimates the proportion of variance explained by one set of variables (e.g., environment) once the influence of other variables (e.g., distance and population structure) has been removed (Legendre and Legendre 2012). Comparing the amount of variance explained by each pRDA with the variance of a model including all explanatory variables (full model) estimates the independent contribution of each set of variables together with the confounded effect induced by collinearity (Peres‐Neto et al. 2006). The explanatory or conditional factors were either environment (organic content and daily temperature range), population structure (summarized by PC1 and PC2 of the population structure PCA based on intergenic SNPs), or distance (PCoA1 and PCoA2 of sea distance). Model significance was tested using the *vegan* *anova.cca* function with 999 permutations (Legendre et al. 2011).

#### 2.4.2 Identification of Climate‐Adapted Eelgrass Meadows—Detection of Putatively Adaptive Outlier Loci and Functional Annotation

As restoration success may be influenced by local adaptation as well as “preadaptation” to future conditions, we used *rdadapt* (Capblancq et al. 2018) to detect outlier loci that were more differentiated in our environmental pRDA model (including daily temperature range and organic content of the sediment and conditioning for genetic and geographic distance) than under a neutral model. We identified outliers using the *rdadapt* function and retaining two RDAs, which rearranges redundant genetic variation associated with environmental variation along composite axes and identifies the loci that are strongly associated with the most important axes. We considered all loci with a *p* value below 0.01 after Bonferroni correction as potential outlier loci. We then used a second independent outlier detection method to identify loci contributing excessively to population differentiation. Using *pcadapt* (version 4.4.0; Luu et al. 2017), we first performed a PCA to identify the PCs that explained a considerable amount of population structure. Second, these PCs were used to assess the correlation between loci and PCs based on the *q* values (*q* < 0.1). Finally, we investigated those loci that were detected by both outlier detection methods in each of the two data sets further and screened the same scaffold as each putative outlier locus of the *Z. marina* genome assembly v.2.1 (Ma et al. 2021) for annotations in physical proximity (up to 15,000 bp distance) to each outlier SNP. Moreover, we plotted the allele frequencies of the outliers detected by both methods in the clone corrected data set according to meadow, and the two strongest environmental variables detected in the RDAs with *ggplot*.

#### 2.4.3 Identification of Climate‐Adapted Eelgrass Meadows—Eelgrass Growth Under Increased Temperature and Decreased Salinity in the Common Garden Mesocosm Experiment

We used the common‐garden mesocosm experiment to test whether the effects of an extreme event (warming and freshening) on eelgrass growth varied among the 10 meadows, and whether those responses varied with provenance (mixed‐ vs. single‐meadow treatments). We used a linear model to evaluate whether meadow, extreme event treatment, provenance strategy, and grazer diversity, and their interactions were significant predictors of growth during the experiment. To explore the relative effect of extreme treatment on growth compared to the control, we calculated the *ln* response ratio (lnRR) of growth between the two treatments across sites (Nakagawa et al. 2023).

## 3 Results

### 3.1 Small‐Scale Environmental Variability

Despite the short sea distances between the 10 meadows (1.5–14 km), we documented clear temperature differences among the meadows (Table S1, Figure 2B; Hattich et al. 2025). The five meadows characterized as “sheltered” experienced higher temperatures, as well as higher variability, than the five “exposed” meadows (Table S1). The recorded daily mean temperatures ranged from 20.54°C to 23.04°C (Table S1). The highest daily maximum temperature was 24.44°C at the sheltered meadow TAN. Importantly, sheltered meadows (color coded in orange on the figures) had a (significantly) higher daily mean temperature than exposed meadows (mean of 22.49°C vs. 20.75°C), a higher daily maximum temperature (23.7°C vs. 21.65°C), and a higher diurnal temperature range (2.29°C vs. 1.66°C; Figure 2B; Wilcoxon test, *p* < 0.001). While we were only able to collect winter temperature data from 5 out of the 10 meadows, those data also showed that winter temperature daily means (4.7°C in sheltered vs. 5.3°C in exposed meadows), maxima (5.3°C vs. 5.7°C), minima (4.0°C vs. 4.9°C), and diurnal temperature range (1.3°C vs. 0.8°C) were significantly different for the two exposed sites compared to the three sheltered sites (Wilcoxon test, *p* < 0.01). In the winter, exposed sites experienced higher temperatures but continued to experience lower daily variability compared to the sheltered meadows (Table S1). Importantly, summer temperatures were strongly (> 0.7) correlated with winter temperatures. Organic content of the sediment ranged from 2.5% to 5.9% (Figure 2C). Although less consistent, the sediment of sheltered meadows typically had a higher organic content (5% vs. 4%; Figure 2C; Table S1).

### 3.2 GOAL 1: Rapid Establishment of Eelgrass

#### 3.2.1 Identification of Eelgrass Meadows With Strong Growth

In our common‐garden mesocosm experiment, eelgrass growth differed significantly among sites, extreme event treatment, and provenance strategy (mixed vs. single meadows), with no significant interactions (Table S4). The sheltered meadows GAS, RAM, and NYC had the highest overall mean growth (Figure S4). The mean growth per site ranged from 32 to 39 cm in the exposed meadows and 31–47 cm in the sheltered meadows. All meadows had large variability in growth (Figure S4).

### 3.3 GOAL 2: Restore Ecosystem‐Services

#### 3.3.1 Identification of Eelgrass Meadows With High Genetic Diversity

To address genetic variability among meadows, we analyzed the genetic dataset consisting of 189 individuals and 20 replicates sampled at the 10 sites and successfully genotyped with 1689 SNPs. After clone correction, leaving only one sample per MLL per site, 111 MLLs genotyped with 1648 polymorphic SNPs remained, with an overall locus missingness of 1.7%. Thirteen MLLs were shared among the meadows of RAM, STY, and TAN, and the “clone corrected” data set therefore contains 124 samples. One MLL was found in seven shoots collected at STY and one shoot in TAN. RAM and TAN shared overall 12 MLLs represented by at least 1 shoot in each meadow. In our main analysis, we focus on this “clone‐corrected” data, but see the supplement for analyses based on all 189 samples.

Genotypic richness differed among meadows with a high number of clones in KOC (*R* = 0.31) and highest genotypic richness at NYC (*R* = 0.83; Figure 2D, Table S5). The percentage of polymorphic loci ranged between 14% and 33% (Figure 2E). There were high levels of private alleles at most meadows ranging from 0 to 84 and indicating their uniqueness (Table S5). There were no strong differences in the genetic diversity estimates for the clone‐corrected data set compared to the data set including all 189 samples (Table S5), apart from the inbreeding coefficient (*F*_IS_), where—as expected—including clones led to a greater deviation from expectations (Meirmans 2024). Moreover, more private alleles were detected when including all samples (mean of 85 vs. 60; Table S5).

The documented environmental differences between exposed and sheltered meadows were also reflected in the genetic diversity assessment (Figure 2D,E): sheltered meadows generally had higher genotypic richness than exposed meadows (mean *R* of sheltered: 0.7; mean *R* exposed: 0.56), a higher polymorphism (mean of 29% vs. 19%) as well as a higher number of private alleles (means of 70 vs. 50).

### 3.4 GOAL 3: Long‐Term Persistence of Restored Eelgrass Meadows

#### 3.4.1 Identification of Locally Adapted Eelgrass Meadows—Genetic Differentiation

There was clear genetic differentiation between exposed and sheltered meadows as well as to some extent sampling location among exposed meadows (Figure 2F). Sheltered meadows clustered more closely to each other and separately from exposed meadows (Figure 2F). When including all samples, clones clustered closely together and were clearly differentiated from the unique genotypes on PC1 and PC2 (Figure S5). *F*_ST_ was overall low across the clone‐corrected data set (0.056) and a Mantel test between sea distance (Table S2) and pairwise *F*_ST_ (Table S3) did not indicate isolation‐by‐distance (Figure S6).

#### 3.4.2 Identification of Locally Adapted Eelgrass Meadows: Genotype–Environment Association Analysis

**FIGURE 3:**
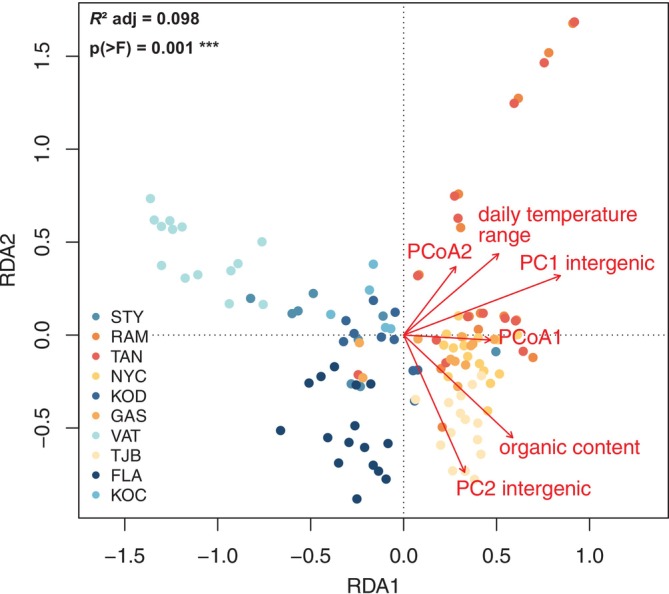
Full RDA (redundancy analysis) including environment, geographic distance (PCoAs), and genetic distance based on principal component analysis using intergenic single nucleotide polymorphisms (SNPs) from 111 eelgrass multilocus lineages (MLLs) and 124 samples genotyped with 1648 SNPs. Each dot represents one MLL sampled at one of the 10 eelgrass meadows along the Swedish west coast. Exposed sites are shown in blues, where increasing darkness indicates sites with decreasing daily temperature range. Sheltered sites are shown in oranges, where increasing darkness indicates increasing daily temperature range (i.e., darkest orange is the site with the highest daily temperature range). Red arrows represent variables that drive the observed population structure. ***p < 0.001.

To investigate whether the observed genetic differentiation between sheltered and exposed meadows is driven by the recorded environmental differences at the sampling sites, we built a full RDA model including the noncorrelating (Figure S2) environmental variables summer diurnal temperature range and organic content of the sediment, as well as the two first PCs of a PCA based on intergenic SNPs for genetic structure (Figure S3) and two PCoAs for sea distance (Figure S1). This model was globally significant and explained 9.8% of the total genetic variance across small‐scale eelgrass populations (Table 1, Figure 3). Genetic distance was identified as the strongest predictor, but organic content, daily temperature range and geographic distance were also significant predictors (Figure 3).

**TABLE 1:** Environmental, geographic, and genetic variables included in the final full RDA (redundancy analysis) and identified as significantly associated with eelgrass genetic variation using forward variable selection.

Variables	*R* ^2^_adj_	Cum *R* ^2^_adj_	*F* value	*p* level
Genetic distance‐PC1_intergenic	0.035	0.032	5.45	**
Genetic distance‐PC2_intergenic	0.023	0.058	3.94	**
Diurnal temperature range	0.017	0.075	3.23	**
Geographic distance—PCoA1	0.008	0.083	2.07	**
Organic content	0.008	0.091	2.00	**
Geographic distance—PCoA2	0.007	0.098	2.01	**

*Note:* Variables were selected through an ordiR2step procedure.

** *p* value between 0.001 and 0.01.

To explore the (statistical) association among descriptors and to better understand the covariation of environmental, spatial, and genetic gradients, we used variance partitioning and observed a high degree of explanatory power in the pRDA models. The effect of geographic distance, genetic structure, and environment were significant even when controlling for the other categories (Table 2). The genetic predictors explained the largest proportion, and each category alone could explain a large proportion (16%–28%) of the explainable variation. Nevertheless, nearly 40% of the explainable genetic variation was confounded among the three different categories (Table 2).

**TABLE 2:** The influence of environment, distance, and genetic structure on genetic variation in eelgrass decomposed with partial redundancy analysis (pRDA).

Partial RDA models	*R* ^2^_adj_	*p* (> *F*)	Proportion of explainable variance
Full model: *F* ~ env. + dist. + struct.	0.098	***	100%
Pure distance: *F* ~ dist. | (env. + struct.)	0.016	***	15.9%
Pure genetic structure: *F* ~ struct. | (env. + dist.)	0.028	***	28.3%
Pure environment: *F* ~ env. | (dist. + struct.)	0.018	***	18.5%

*Note:* The proportion of explainable variance represents the total constrained variation explained by the full model. env. stands for the environmental parameters daily temperature range and organic content. dist. stands for the two PCoAs representing geographic distance. struct. stands for PC1 and PC2 of genetic distance based on intergenic SNPs.

*** *p* value below 0.001.

When basing RDA and pRDA analyses on all 189 samples, rather than the clone‐corrected data, the same environmental parameters were selected (Figure S7, Table S6). However, the PCA showed clonal replicates clustering closely together and differentiating from unique genotypes (Figure S5). This has downstream effects on the RDA and pRDA analyses, which clearly point to genetic differentiation as the most important driver of allele frequencies (Figure S7; Tables S6 and S7).

#### 3.4.3 Identification of Climate‐Adapted Eelgrass Meadows—Putatively Adaptive Outlier Loci

Given the genetic differentiation between sheltered and exposed meadows, and because the local environment is a significant driver of this differentiation, we aimed to identify putative adaptive loci. Using *rdadapt*, we identified 112 putative adaptive loci, which have allele frequencies that showed an association with daily temperature range and/or organic content of the sediment in the clone‐corrected data set and 218 putative outliers in the data set based on all 189 samples (Figure S8). *Pcadapt* identified 30 and 13 loci, and 22 and 11 outliers were identified by both methods, respectively. We were able to annotate approximately half of the putative adaptive loci (Tables S8 and S9). The allele frequencies of one of the strongest outlier loci (Chr05_26498677)—annotated as *putative Auxin response factor*—showed a very clear association with daily temperature variability (Figure 2G), as did three more loci annotated as *Acetyl‐CoA oxidase* (Chr01_25714157), *Thaumatin‐like protein 1* (Chr01_18329242) and one that was assigned into an assembly gap (Chr01_18516104; Figure S9).

#### 3.4.4 Identification of Climate‐Adapted Eelgrass Meadows—Eelgrass Growth Under an Extreme Event

In the final step, we investigated whether there were also phenotypic differences in growth under control versus extreme event conditions. In our common‐garden experiment, eelgrass growth was significantly different among sites, provenance strategy, and extreme event treatment with no statistically significant interactions (Table S4). As there was no interaction between site and provenance (Table S4; Figure S10), we investigated the impact of the extreme event treatment after combining data on both provenance strategies within site.

**FIGURE 4:**
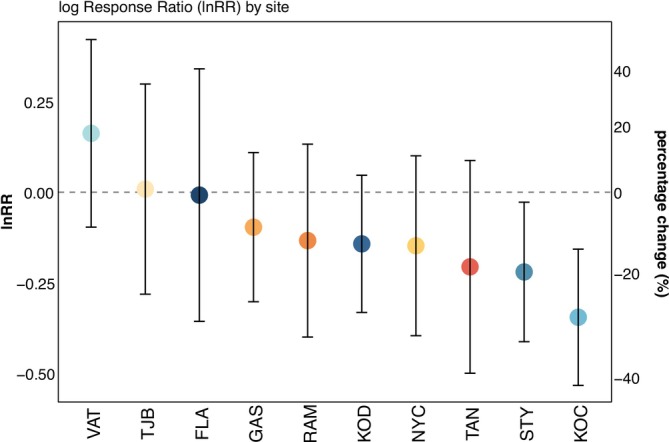
Log response ratio (lnRR) of growth between control and extreme event treatment across meadows, representing the magnitude and direction of the treatment effect on growth. Positive lnRR values indicate a beneficial treatment effect, whereas negative values suggest a detrimental effect. The error bars represent the 95% confidence interval associated with the lnRR estimates, providing a measure of variability in the response. The sites are ordered according to effect size. The n per category is 42–48. Exposed sites are shown in blues, where increasing darkness indicates sites with decreasing daily temperature range. Sheltered sites are shown in oranges, where increasing darkness indicates increasing daily temperature range (i.e., darkest orange is the site with the highest daily temperature range).

The marine heatwave and freshening event treatment had a mean temperature elevation of 3.7°C and a 4 psu reduction compared to the control treatment (Figures S11 and S12). Over the 9 days of the experiment, the control treatment experienced a mean temperature of 21.8°C and a mean salinity of 24 psu, while the extreme event treatment experienced a mean of 25.5°C and 20 psu across diurnal and daily temperature fluctuations (Figures S11 and S12). The effect of the extreme event treatment had a different magnitude in response and variability on growth across meadows (Figure 4, see also Figure S10). The strongest negative effects of the extreme event, simulating a marine heatwave with concurrent freshening, were observed at KOC, STY, and TAN (Figure 4).

## 4 Discussion

Seagrasses are among the most productive, yet highly threatened ecosystems on earth (Waycott et al. 2009). Legislation for protection and restoration of seagrass habitats has been established in many nations (van Katwijk et al. 2016), culminating in Europe in the acceptance of the EU Nature Restoration Law in 2024, which lists seagrasses as a key habitat (EU 2024). A large number (1786 in 2015) of seagrass restoration initiatives have been carried out globally over the last decades, but the estimated survival rate is low (van Katwijk et al. 2016). The combination of factors that contribute to successful restoration remain poorly understood. Emphasis has been given to restoration site selection and the reversal of environmental degradation to conditions conducive for seagrass growth (van Katwijk et al. 2016). Moreover, restoration effort scale has been shown to be an important predictor for success, with larger and more densely planted restored areas performing better (van Katwijk et al. 2016). While these aspects are crucial components for successful seagrass restoration, we additionally want to highlight here that the choice of donor material also likely influences restoration success. Environment, phenotype, and genetics can vary considerably on small spatial scales and can support the selection of donor material to increase the likelihood of achieving the three main goals of (1) rapid establishment, (2) high ecosystem functioning, and (3) long‐lasting restoration success (Figure 1). Given the higher restoration success when using local donor material due to local adaptation and/or better physiological condition (van Katwijk et al. 2016) and to harness the benefits of mixed and predictive provenancing (Bailey et al. 2021), fine‐scale genetic assessments around every potential future restoration meadow would be ideal. Striving for the three conceptual goals of restoration (Figure 1), we provide here an example of how genetic, phenotypic, and environmental information could be used to design a restoration program that aims to identify local mixed donor materials that are likely to lead to rapid establishment, the return of ecosystem functioning and which may enable the use of preadapted genotypes in order to future‐proof restoration programs (Wood et al. 2020, 2021).

### 4.1 On the Way to Achieve GOAL 1 of Rapid Establishment

While the terrestrial plant literature discusses the straight link between strong initial growth with rapid establishment success (e.g., Kettenring et al. 2014), this aspect has, to our knowledge, received very little attention in the seagrass restoration literature, despite clear differences in survival among different donor sites (Eriander et al. 2016; van Katwijk et al. 1998), and large variation in eelgrass growth across the Northern hemisphere (Duffy et al. 2022). For eelgrass, the most commonly used techniques are the use of plugs (cores of sediment and shoots), the transplantation of shoots with bare roots and rhizomes, with and without anchoring, and restoration using seeds (Eriander et al. 2016; Orth et al. 2012; van Katwijk et al. 2016). In Sweden, shoot transplantations have seen the highest success in restoration efforts (Eriander et al. 2016, 2025). In salt marshes, this technique has been shown to lead to higher genetic diversity and to increased resilience of restored marshes (Crosby et al. 2024). Given the observed differences in eelgrass growth form (Duffy et al. 2022), guidelines for eelgrass restoration have generally suggested that donor plants should “look” as similar as possible to the plants being restored (Eriander et al. 2016; M. Fonseca and Bell 1998; Olsen et al. 2014; van Katwijk et al. 2009). In our common‐garden experiment, which simulated semi‐natural conditions in mesocosm tanks, growth was significantly different among sites and generally higher in donor materials from sheltered meadows. In our short‐term experiment, we assessed growth, which seems a good proxy for the emergence of site shoots and therefore likelihood of expansion at the restoration site (Figure S3). However, for actual restoration trials that require monitoring over longer time frames, shoot density or the emergence of side‐shoots would be a more directly relevant measure of restoration success.

Finally, an interesting observation is that the three exposed sites with the lowest genotypic richness (i.e., highest number of clones) showed the least variability in growth, which could indicate that an extent of variability in growth has a genetic basis, and identical genotypes perform more similar to each other than unique genotypes.

### 4.2 On the Way to Achieve GOAL 2: The Restoration of Ecosystem Functioning

In general, higher genetic diversity is associated with higher resistance and resilience to disturbances and is acknowledged to increase the chances that populations will evolve in response to changes in climate (Jump et al. 2009; Reusch et al. 2005). In seagrass restoration in particular, the role of genetic diversity in the success of restoration and the return of ecosystem services has been established for several decades (Evans et al. 2017; Procaccini and Piazzi 2001; Reynolds, McGlathery, et al. 2012; Tomas et al. 2011). In our analysis, there were clear differences in genetic diversity among meadows. Genotypic richness differed between 0.31 and 0.83 among the meadows assessed at this small‐spatial scale. The percentage of polymorphic alleles also varied considerably from 15% to 32% and was consistently higher in sheltered meadows. These differences in genetic diversity at the micro‐habitat scale are comparable to differences observed at the scale of 100 s km (Jahnke et al. 2018; Ries et al. 2023; Faust et al. 2025). Given very low estimates of effective population size and high inbreeding values of eelgrass meadows in the area (Faust et al. 2025), it is prudent to assume that eelgrass meadows may have less genetic variation to adapt to environmental change than is predicted from locally high abundance (Sotka et al. 2024). Whenever possible, we therefore suggest selecting donor meadows with high genotypic and genetic diversity, always considering genetic structure in the area.

### 4.3 On the Way to Achieve GOAL 3: The Long‐Term Persistence of Restored Areas

The important role of local adaptation in seagrass restoration has been shown in several cases (Jahnke et al. 2015; Reynolds, Waycott, et al. 2012), may partly explain the generally low restoration success rate (DuBois et al. 2022; van Katwijk et al. 2016) and has led to the recommendation to source donor material from the same genetic cluster as the restoration site (e.g., Eriander et al. 2025; Jones 2003). Our understanding of how to select donor sites and genotypes for restoration to promote resilience to climate change is still developing (Adam 2019; Crosby et al. 2024), but the component of future‐proofing seagrass restoration is being addressed increasingly (Nimbs et al. 2024; Pazzaglia et al. 2021). Mixed and predictive provenancing (Breed et al. 2019), that is, selecting donor material from several meadows and considering preadaptive traits to future environmental conditions, may be the only way to ensure long‐term success of restorations. Nevertheless, moving species or genotypes outside their current ranges (assisted migration) or beyond the scale of natural dispersal (assisted gene flow) is also controversial as it might come with the risk of outbreeding depression, loss of local adaptation, and disease transmission (Aitken et al. 2024; Aitken and Whitlock 2013; Govers et al. 2016; Jakobsson‐Thor et al. 2020; Weeks et al. 2011). We therefore investigated here, if preadapted genotypes can also be found at the local scale that is within the dispersal scale of eelgrass (Jahnke et al. 2018, 2020) for three aspects.

#### 4.3.1 Goal 3: 1. Local Adaptation to Meadow Environment

Local adaptation is common in plant species (Capblancq et al. 2023). In our small‐spatial scale analysis, there was no evidence of isolation‐by‐distance, but instead a clear genetic differentiation by habitat‐type between exposed and sheltered meadows. The RDA further confirmed the significant and strong role of environment (temperature and sediment characteristics, both also driven by exposure) in genetic variability and therefore local adaptation on this small spatial scale. “Type‐matching” (van Katwijk et al. 2016) of exposed donor materials at an exposed restoration site, and sheltered donor materials at a sheltered restoration site, seems therefore advisable as the use of local type‐matching plants may be beneficial due to the presence of locally adapted loci or gene complexes (M. S. Fonseca 2011; Hämmerli and Reusch 2002; Jahnke et al. 2015; Sinclair et al. 2013).

#### 4.3.2 Goal 3: 2. Climate–Adaptation—Putative Adaptive Loci

Given the clear genetic differences among sheltered and exposed meadows, we explored the presence of putative adaptive outlier loci. While most fitness‐related traits in plants have a polygenic basis (Falke et al. 2013), the presence of putative adaptive loci, which may confer preadaptation, is a prerequisite for applying predictive provenancing in restoration (Nimbs et al. 2024). In our analysis, we identified one locus that has a clear association with daily temperature range at the site. This putative adaptive locus on chromosome 5 (Chr05_26498677; Figure 2G) has a higher frequency of the most common allele in sheltered meadows. Our analysis therefore indicates that genotypes with this allele may be genetically adapted to a warmer and more variable temperature regime, which they experience in the more sheltered habitat. These meadows may therefore represent reservoirs of heat‐tolerant genotypes and phenotypes (DuBois et al. 2022), and harbor donor material that is more likely to survive the high temperatures that all meadows are predicted to experience. This locus is annotated as *putative Auxin response factor*, which has an important role in plant growth and development (Guilfoyle and Hagen 2007), and has also been identified as an outlier in a large‐scale genetic analysis of eelgrass.

Three more loci were also detected as putatively adaptive with a similar distinction in allele frequencies between sheltered and exposed meadows (Figure S9). The gene functions associated with the two loci with annotations are *Acetyl‐CoA oxidase* and *Thaumatin‐like protein 1*, which also suggest involvement in hormonal regulation of plant development and growth, as well as pathogen defence. Other annotations among the 33 outlier loci identified with both outlier detection methods also suggest possible involvement in key regulatory processes of plant development, death and stress response, and include *WALLS ARE THIN 1*, *BONZAI 1*, *NHL repeat‐containing protein*, *Pectinesterase*, and *MACPF domain‐containing protein*. Most of the 17 possible annotations that we found for the 33 outlier loci belong to proteins that were also differentially expressed in studies on transcriptomic adaptation to temperature in eelgrass in the North‐East Atlantic (Jueterbock et al. 2016, 2021) and include several *(putative) Auxin response factors*, *Pectinesterases*, *Acyl‐CoA oxidases*, *Thaumatin‐like protein 1*, *Prefoldin subunits*, *myb domain proteins 15*, *NHL repeat‐containing proteins*, *MACPF domain‐containing proteins*, *WALLS ARE THIN 1*, *GTPase HflX*, *CASP‐like protein*, *signal peptide peptidase SppA 67K type*, *Prefoldin subunit*, *Adenosylmethionine decarboxylase*, and *BONZAI 1*. However, none of our 33 outlier loci were found in recent genotype–environment association studies in the Pacific (Schiebelhut et al. 2023) and North‐West Atlantic (Sotka et al. 2024).

#### 4.3.3 Goal 3: 3. Climate–Adaptation—Difference in Growth Under Extreme Event

Goal 3 for achieving long‐term persistence of restored areas entails that the donor material performs well under both current and future climates. Hence, to understand the ability of the donor material to persist and thrive under future conditions, we should test it under potential future climates (Nadeau et al. 2024). As this implies either long‐term monitoring in the field or long‐term experiments, extreme events are often used alternatively as proxies for the future (Nadeau et al. 2024). In our experiment, growth was negatively affected by the extreme event treatment, simulating a marine heatwave with concurrent freshening, in 7 out of the 10 tested eelgrass meadows. It would be prudent for any putative restoration efforts in the area to avoid using donor material from the three meadows (STY, TAN, and KOC) that were most negatively impacted by the extreme event, and which also have low allele frequencies of the putative adaptive locus Chr05_26498677 in the context of the exposure they experience.

### 4.4 Recommendations for Restoration

There are still a few genomic studies of eelgrass at this small scale, but the results indicate that genetic differentiation on the micro‐habitat scale is prevalent (this study; Schiebelhut et al. 2023; Sotka et al. 2024). Aiming to preserve valuable eelgrass meadows will require proactive conservation and restoration efforts, and we exemplify here how eelgrass restoration could benefit from considering the principles of restoration genomics when sourcing donor material. For any new restoration action, we would propose to follow previously suggested guidelines and assess and monitor sediment and temperature characteristics at the selected restoration site, and any potential donor meadows (Moksnes et al. 2016). In general, donor materials should then be selected from meadows with similar environmental characteristics. If feasible, mesocosm or field experiments could then determine potential donor meadows with strong growth under current and future/extreme conditions.

Understanding where eelgrass is adapted to local and future environmental conditions is key for identifying optimal donor materials (Nimbs et al. 2024). If genomic data are available or can be generated, meadows with low clonality, high genetic diversity and with allele frequencies of putative adaptive alleles at a desirable level for the currently observed and/or modeled future environment at the restoration site, can be selected to boost resilience to future climate. Despite no evidence of consistent advantage of a mixed‐provenance strategy within site in the short‐term common‐garden mesocosm experiments, we would recommend a strategy of “regional admixture provenancing” (Bucharova et al. 2019). Given the overall small‐scale variability, mixed and predictive provenancing on the local scale has the highest likelihood to identify donor materials that reduces the risks of outbreeding and inbreeding depression, maladaptation, and genetic pollution (Coleman et al. 2020), while increasing resilience of the managed system through the portfolio effect (Nadeau et al. 2024).

With regard to the region of the assessment in particular, the Swedish guidelines for eelgrass restoration have just been updated (Eriander et al. 2025) and include an entire chapter on genetic diversity and connectivity. While restoration practices are under continuing development as more data comes in, the current guidelines for the Swedish west coast are to choose a restoration site based on habitat characteristics suitable for eelgrass growth and the possibility to increase connectivity between meadows. For sourcing donor materials, the recommendation is to use a donor site that is from the same genetic cluster and has high genetic diversity. If genetic data are lacking, the recommendation is to source from large eelgrass meadows, as they tend to have higher genetic diversity, a larger effective population size and lower inbreeding values (Faust et al. 2025). These sound recommendations are based on genetic studies and eelgrass mapping at the scale of 10s–100s km. Our data indicate that it is desirable to additionally include environmental aspects and the possibility of small‐scale local adaptation when selecting donor materials.

If the overall aim is to not only restore the restoration site but allow gene flow and support natural recovery, it seems advisable to choose relatively exposed sites for restoration (Eriander et al. 2025). As we observed strong genetic differentiation between exposed and sheltered sites, and as a considerable amount of the genetic variation is explained by environment even on this small spatial scale, we recommend sourcing donor material for an exposed restoration site from exposed donor meadows with similar environmental variability.

With regard to our specific study area in the Koster archipelago, we propose to source donor material that has the highest likelihood to lead to rapid establishment, maximizes the return of ecosystem functioning, and which may enable the selection of preadapted genotypes in order to future‐proof restoration programs (Figure 1). As the three goals are to some extent mutually exclusive, and as there are clear differences in environment, genetic differentiation, and genetic diversity, we recommend mixed and predictive provenancing on the local scale, and considering exposure in the first instance. None of the genetic and phenotypic aspects we assessed seem to be a good proxy for achieving all three goals, but by assessing several aspects classified under the three goals of restoration (Figure 1), we were able to narrow down the pool of potential donor meadows to a small selection of meadows that hold the biggest promise for achieving all three goals of restoration (Box 1). For restoring an exposed site in the Koster Sea, we would recommend the use of local admixed donor material from the exposed sites VAT and FLA and the sheltered TJB to aim for high genetic diversity, high local adaptation, high likelihood for the presence of preadapted alleles, strong growth, and plants that can withstand future conditions without negative impact on growth (Box 1). For the restoration of a sheltered site, we would recommend donor material from NYC and TJB for the highest likelihood of reaching all three goals of restoration (Box 1). We would also recommend avoiding sourcing donor material from three of the meadows (STY, TAN, and KOC), as these meadows have high clonality or low overall growth and showed a strong decrease in growth under the extreme event treatment in the common garden mesocosm experiments.

Our results highlight that eelgrass is locally adapted to past and present environmental conditions on small spatial scales. This knowledge can be used to improve the sourcing of donor materials for improved restoration success depending on the environmental conditions at the restoration site. We propose that our assessment can provide the beginning of a donor registry (Nimbs et al. 2024), which could provide a first step to incorporate evolutionary aspects in the selection of donor material for improving restoration success and future proofing restoration. Biodiversity conservation requires conserving the evolutionary potential (Aitken et al. 2024). The ability to consciously select donor materials with desirable genetic and phenotypic characteristics and planning for “future proofing” restoration will likely play an important role in maintaining and restoring eelgrass meadows under climate change. Successful eelgrass restoration with genetically diverse and resilient phenotypes may also have cascading impacts on ecosystem function and community dynamics (DuBois et al. 2022; Hughes and Stachowicz 2009).

BOX 1 **Beginning of a donor registry to narrow down the pool of potential donor meadows to those meadows that hold the biggest promise for achieving all three goals of restoration.**

We recommend mixed and predictive provenancing at the local scale. We used the assessment of 10 meadows to select 2 and 3 meadows as donor materials for sheltered and exposed restoration sites, respectively (names highlighted in bold) to aim for high genetic diversity, high local adaptation, high likelihood for the presence of preadapted alleles, high growth and plants that can withstand future conditions without negative impact on growth. Highlighted in green are sites that have the highest likelihood to achieve a specific goal. Orange indicates a medium likelihood, that is, sites that may also be suitable for achieving a specific goal, and red indicates sites with a low potential to satisfy a certain goal. *T*_Diff_ stands for daily temperature range; *R* for genotypic richness, and Pol_MLL stands for the percentage of polymorphic loci in the clone corrected data set.

**Table:** 

Site	Exposure	*T*_Diff_ (°C)	Organic content (%)	*R*	Pol_MLL (%)	Allele freq of Chr5	Mean growth	Response ratio extreme event	Donor site for goal 1—growth	Donor site for goal 2—genetic diversity	Donor site for goal 3—persistence and adaptive potential
STY 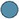	EXP	1.67	3.5	0.42	22	0.39	35.85	−0.22		High clonality	Extreme event has a negative effect on growth
KOD 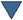	EXP	1.53	5.2	0.5	20	0.6	38.05	−0.14	Exposed site with high mean growth		
**VAT** 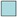	EXP	1.95	2.5	0.76	21	0.25	39.47	0.16	Exposed site with high mean growth	Exposed site with high genetic diversity	Exposed site with low impact of extreme event on growth
**FLA** 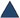	EXP	1.36	5.5	0.79	22	0.63	31.98	−0.01		Exposed site with highest genetic diversity	Exposed site with highest frequency of putative adaptive locus and low impact of extreme event on growth
KOC 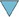	EXP	1.79	4.5	0.31	15	0.5	32.08	−0.35		High clonality and lowest genetic diversity	Extreme event has a negative effect on growth
RAM 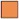	SHELT	*2.69*	4.5	0.68	23	0.88	44.58	−0.13	Sheltered site with high mean growth		
TAN 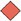	SHELT	2.69	4.1	0.68	24	0.79	30.85	−0.21	Lowest overall growth		Extreme event has a negative effect on growth
**NYC** 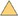	SHELT	2.06	5.3	0.83	32	0.84	37.57	−0.15	Sheltered site with high mean growth	Sheltered site with high genetic diversity	Sheltered site with high frequency of putative adaptive locus
GAS 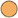	SHELT	2.44	4.9	0.53	25	0.86	47.05	−0.10	Sheltered site with high mean growth	High clonality	
**TJB** 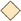	SHELT	1.96	5.9	0.78	30	0.97	37.15	0.01	Sheltered site with high mean growth	Sheltered site with high genetic diversity	Sheltered site with highest frequency of putative adaptive locus and low impact of extreme event on growth

## Conflicts of Interest

The authors declare no conflicts of interest.

## Supporting information


Data S1.

## Data Availability

The environmental data, genetic vcf files, and R scripts are available at https://doi.org/10.17044/scilifelab.29128325. Sequence data for this study are available on NCBI in BioProject: PRJNA1043091.

## References

[eva70127-bib-0001] Abbott, J. M. , and J. J. Stachowicz . 2016. “The Relative Importance of Trait vs. Genetic Differentiation for the Outcome of Interactions Among Plant Genotypes.” Ecology 97, no. 1: 84–94. 27008778 10.1890/15-0148.1

[eva70127-bib-0002] Adam, P. 2019. “Salt Marsh Restoration.” In Coastal Wetlands. https://www.sciencedirect.com/science/article/pii/B978044463893900023X. Elsevier.

[eva70127-bib-0003] Agneray, A. C. , T. L. Parchman , and E. A. Leger . 2022. “Phenotypes and Environment Predict Seedling Survival for Seven Co‐Occurring Great Basin Plant Taxa Growing With Invasive Grass.” Ecology and Evolution 12, no. 5: e8870. 35509617 10.1002/ece3.8870 PMC9055296

[eva70127-bib-0004] Aitken, S. N. , R. Jordan , and H. R. Tumas . 2024. “Conserving Evolutionary Potential: Combining Landscape Genomics With Established Methods to Inform Plant Conservation.” Annual Review of Plant Biology 75, no. 1: 707–736. 10.1146/annurev-arplant-070523-044239 38594931

[eva70127-bib-0005] Aitken, S. N. , and M. C. Whitlock . 2013. “Assisted Gene Flow to Facilitate Local Adaptation to Climate Change.” Annual Review of Ecology, Evolution, and Systematics 44, no. 1: 367–388.

[eva70127-bib-0006] Arnaud‐Haond, S. , C. M. Duarte , F. Alberto , and E. A. Serrão . 2007. “Standardizing Methods to Address Clonality in Population Studies.” Molecular Ecology 16, no. 24: 5115–5139. 17944846 10.1111/j.1365-294X.2007.03535.x

[eva70127-bib-0007] Baden, S. , C. Boström , S. Tobiasson , H. Arponen , and P.‐O. Moksnes . 2010. “Relative Importance of Trophic Interactions and Nutrient Enrichment in Seagrass Ecosystems: A Broad‐Scale Field Experiment in the Baltic−Skagerrak Area.” Limnology and Oceanography 55, no. 3: 1435–1448.

[eva70127-bib-0008] Baden, S. , M. Gullström , B. Lundén , L. Pihl , and R. Rosenberg . 2003. “Vanishing Seagrass ( *Zostera marina* , L.) in Swedish Coastal Waters.” Ambio 32, no. 5: 374–377. 14571969 10.1579/0044-7447-32.5.374

[eva70127-bib-0009] Bailey, T. G. , P. A. Harrison , N. J. Davidson , et al. 2021. “Embedding Genetics Experiments in Restoration to Guide Plant Choice for a Degraded Landscape With a Changing Climate.” Ecological Management & Restoration 22, no. S2: 92–105.

[eva70127-bib-0010] Bjornstad, O. N. 2022. “Spatial Covariance Functions [R Package ncf Version 1.3‐2].” Comprehensive R Archive Network (CRAN). https://CRAN.R‐project.org/package=ncf.

[eva70127-bib-0011] Boström, C. , S. Baden , A.‐C. Bockelmann , et al. 2014. “Distribution, Structure and Function of Nordic Eelgrass ( *Zostera marina* ) Ecosystems: Implications for Coastal Management and Conservation.” Aquatic Conservation: Marine and Freshwater Ecosystems 24, no. 3: 410–434. 26167100 10.1002/aqc.2424 PMC4497458

[eva70127-bib-0012] Boyd, P. W. , S. Collins , S. Dupont , et al. 2018. “Experimental Strategies to Assess the Biological Ramifications of Multiple Drivers of Global Ocean Change‐A Review.” Global Change Biology 24, no. 6: 2239–2261. 29476630 10.1111/gcb.14102

[eva70127-bib-0013] Boyd, P. W. , C. E. Cornwall , A. Davison , et al. 2016. “Biological Responses to Environmental Heterogeneity Under Future Ocean Conditions.” Global Change Biology 22, no. 8: 2633–2650. 27111095 10.1111/gcb.13287

[eva70127-bib-0014] Boyé, A. , O. Gauthier , R. Becheler , et al. 2022. “Drivers and Limits of Phenotypic Responses in Vulnerable Seagrass Populations: *Zostera marina* in the Intertidal.” Journal of Ecology 110, no. 1: 144–161.

[eva70127-bib-0015] Breed, M. F. , P. A. Harrison , C. Blyth , et al. 2019. “The Potential of Genomics for Restoring Ecosystems and Biodiversity.” Nature Reviews. Genetics 20, no. 10: 615–628. 10.1038/s41576-019-0152-0 31300751

[eva70127-bib-0016] Bucharova, A. , O. Bossdorf , N. Hölzel , J. Kollmann , R. Prasse , and W. Durka . 2019. “Mix and Match: Regional Admixture Provenancing Strikes a Balance Among Different Seed‐Sourcing Strategies for Ecological Restoration.” Conservation Genetics 20, no. 1: 7–17.

[eva70127-bib-0017] Capblancq, T. , and B. R. Forester . 2021. “Redundancy Analysis: A Swiss Army Knife for Landscape Genomics.” Methods in Ecology and Evolution 12, no. 12: 2298–2309.

[eva70127-bib-0018] Capblancq, T. , S. Lachmuth , M. C. Fitzpatrick , and S. R. Keller . 2023. “From Common Gardens to Candidate Genes: Exploring Local Adaptation to Climate in Red Spruce.” New Phytologist 237, no. 5: 1590–1605. 36068997 10.1111/nph.18465 PMC10092705

[eva70127-bib-0019] Capblancq, T. , K. Luu , M. G. B. Blum , and E. Bazin . 2018. “Evaluation of Redundancy Analysis to Identify Signatures of Local Adaptation.” Molecular Ecology Resources 18, no. 6: 1223–1233. 29802785 10.1111/1755-0998.12906

[eva70127-bib-0020] Coleman, M. A. , G. Wood , K. Filbee‐Dexter , et al. 2020. “Restore or Redefine: Future Trajectories for Restoration.” Frontiers in Marine Science 7: 237.

[eva70127-bib-0021] Combrink, C. A. , R. Henriques , M. J. Jackson , and S. von der Heyden . 2024. “Conservation Implications of Strong Population Structure Despite Admixture in an Endangered African Seagrass.” Aquatic Conservation: Marine and Freshwater Ecosystems 34, no. 12: e70012. 10.1002/aqc.70012.

[eva70127-bib-0022] Crosby, S. C. , D. M. Hudson , A. R. Hughes , et al. 2024. “Structure and Function of Restored and Natural Salt Marshes: Implications for Ecosystem Resilience and Adaptive Potential.” Estuaries and Coasts: Journal of the Estuarine Research Federation 47, no. 6: 1561–1578.

[eva70127-bib-0023] Danecek, P. , A. Auton , G. Abecasis , et al. 2011. “The Variant Call Format and VCFtools.” Bioinformatics 27, no. 15: 2156–2158. 21653522 10.1093/bioinformatics/btr330 PMC3137218

[eva70127-bib-0024] Dauphin, B. , C. Rellstab , R. O. Wüest , et al. 2023. “Re‐Thinking the Environment in Landscape Genomics.” Trends in Ecology & Evolution 38, no. 3: 261–274. 36402651 10.1016/j.tree.2022.10.010

[eva70127-bib-0025] Dorken, M. E. , and C. G. Eckert . 2001. “Severely Reduced Sexual Reproduction in Northern Populations of a Clonal Plant, *Decodon verticillatus* (Lythraceae).” Journal of Ecology 89, no. 3: 339–350.

[eva70127-bib-0026] Dormann, C. F. , J. Elith , S. Bacher , et al. 2013. “Collinearity: A Review of Methods to Deal With It and a Simulation Study Evaluating Their Performance.” Ecography 36, no. 1: 27–46.

[eva70127-bib-0027] DuBois, K. , K. N. Pollard , B. J. Kauffman , S. L. Williams , and J. J. Stachowicz . 2022. “Local Adaptation in a Marine Foundation Species: Implications for Resilience to Future Global Change.” Global Change Biology 28, no. 8: 2596–2610. 35007376 10.1111/gcb.16080

[eva70127-bib-0028] Duffy, J. E. , J. J. Stachowicz , P. L. Reynolds , et al. 2022. “A Pleistocene Legacy Structures Variation in Modern Seagrass Ecosystems.” Proceedings of the National Academy of Sciences of the United States of America 119, no. 32: e2121425119. 35914147 10.1073/pnas.2121425119 PMC9371661

[eva70127-bib-0029] Eriander, L. , B. Alenius , I. E , A. Olsson , and P.‐O. Moksnes . 2025. Handbok för Restaurering av Ålgräs i Sverige. Uppdatering av Kunskapsläget 2016‐2024 [Rapport Nummer 2025]. Havs Och Vattenmyndigheten.

[eva70127-bib-0030] Eriander, L. , E. Infantes , M. Olofsson , J. L. Olsen , and P.‐O. Moksnes . 2016. “Assessing Methods for Restoration of Eelgrass ( *Zostera marina* L.) in a Cold Temperate Region.” Journal of Experimental Marine Biology and Ecology 479: 76–88.

[eva70127-bib-0031] Eriander, L. , K. Laas , P. Bergström , L. Gipperth , and P.‐O. Moksnes . 2017. “The Effects of Small‐Scale Coastal Development on the Eelgrass ( *Zostera marina* L.) Distribution Along the Swedish West Coast—Ecological Impact and Legal Challenges.” Ocean and Coastal Management 148: 182–194.

[eva70127-bib-0032] Eriksson, B. K. , U. Bergström , L. L. Govers , and J. S. Eklöf . 2023. “Trophic Cascades in Coastal Ecosystems.” In Reference Module in Earth Systems and Environmental Sciences. Elsevier.

[eva70127-bib-0033] EU . 2024. “Regulation (EU) 2024/1991 of the European Parliament and of the Council of 24 June 2024 on Nature Restoration and Amending Regulation (EU) 2022/869.” https://eur‐lex.europa.eu/legal‐content/EN/TXT/HTML/?uri=OJ:L_202401991.

[eva70127-bib-0034] Evans, S. M. , A. Vergés , and A. G. B. Poore . 2017. “Genotypic Diversity and Short‐Term Response to Shading Stress in a Threatened Seagrass: Does Low Diversity Mean Low Resilience?” Frontiers in Plant Science 8: 1417. 28855915 10.3389/fpls.2017.01417 PMC5557787

[eva70127-bib-0035] Falke, K. C. , S. Glander , F. He , J. Hu , J. de Meaux , and G. Schmitz . 2013. “The Spectrum of Mutations Controlling Complex Traits and the Genetics of Fitness in Plants.” Current Opinion in Genetics & Development 23, no. 6: 665–671. 24268985 10.1016/j.gde.2013.10.006

[eva70127-bib-0036] Faust, E. , K. Rigby , A. Olsson , B. Alenius , P.‐O. Moksnes , and M. Jahnke . 2025. “Empowering Regional Conservation: Genetic Diversity Assessments as a Tool for Eelgrass Management.” Molecular Ecology: e17656. 10.1111/mec.17656. 39801017 PMC12684364

[eva70127-bib-0037] Fonseca, M. , and S. Bell . 1998. “Influence of Physical Setting on Seagrass Landscapes Near Beaufort, North Carolina, USA.” Marine Ecology Progress Series 171: 109–121.

[eva70127-bib-0038] Fonseca, M. S. 2011. “Addy Revisited: What has Changed With Seagrass Restoration in 64 Years?” Ecological Restoration 29: 73–81. https://er.uwpress.org/content/29/1‐2/73.short.

[eva70127-bib-0039] Gaeckle, J. L. , and F. Short . 2002. “A Plastochrone Method for Measuring Leaf Growth in Eelgrass, *Zostera marina* L.” Bulletin of Marine Science 71: 1237–1246.

[eva70127-bib-0040] Goudet, J. 2005. “Hierfstat, a Package for r to Compute and Test Hierarchical *F*‐Statistics.” Molecular Ecology Notes 5, no. 1: 184–186.

[eva70127-bib-0041] Govers, L. L. , W. A. Man In't Veld , J. P. Meffert , et al. 2016. “Marine *Phytophthora* Species Can Hamper Conservation and Restoration of Vegetated Coastal Ecosystems.” Proceedings. Biological Sciences 283, no. 1837: 20160812. 10.1098/rspb.2016.0812. 27559058 PMC5013788

[eva70127-bib-0042] Guilfoyle, T. J. , and G. Hagen . 2007. “Auxin Response Factors.” Current Opinion in Plant Biology 10, no. 5: 453–460. 17900969 10.1016/j.pbi.2007.08.014

[eva70127-bib-0043] Hämmerli, A. , and T. B. H. Reusch . 2002. “Local Adaptation and Transplant Dominance in Genets of the Marine Clonal Plant *Zostera marina* .” Marine Ecology Progress Series 242: 111–118.

[eva70127-bib-0044] Hattich, G. S. I. , M. Jahnke , S. Enge , et al. 2025. “Small‐Scale Thermal Habitat Variability May Not Determine Seagrass Resilience to Climate Change.” *Limnology and Oceanography*, Early View.

[eva70127-bib-0045] Hays, C. G. , T. C. Hanley , A. R. Hughes , S. B. Truskey , R. A. Zerebecki , and E. E. Sotka . 2021. “Local Adaptation in Marine Foundation Species at Microgeographic Scales.” Biological Bulletin 241, no. 1: 16–29. 34436968 10.1086/714821

[eva70127-bib-0046] Hemstrom, W. , and M. Jones . 2023. “snpR: User Friendly Population Genomics for SNP Data Sets With Categorical Metadata.” Molecular Ecology Resources 23, no. 4: 962–973. 36239472 10.1111/1755-0998.13721

[eva70127-bib-0047] Hughes, A. R. , B. D. Inouye , M. T. J. Johnson , N. Underwood , and M. Vellend . 2008. “Ecological Consequences of Genetic Diversity.” Ecology Letters 11, no. 6: 609–623. 18400018 10.1111/j.1461-0248.2008.01179.x

[eva70127-bib-0048] Hughes, A. R. , and J. J. Stachowicz . 2004. “Genetic Diversity Enhances the Resistance of a Seagrass Ecosystem to Disturbance.” Proceedings of the National Academy of Sciences of the United States of America 101, no. 24: 8998–9002. 15184681 10.1073/pnas.0402642101 PMC428461

[eva70127-bib-0049] Hughes, A. R. , and J. J. Stachowicz . 2009. “Ecological Impacts of Genotypic Diversity in the Clonal Seagrass *Zostera marina* .” Ecology 90, no. 5: 1412–1419. 19537560 10.1890/07-2030.1

[eva70127-bib-0050] Jahnke, M. , P. de Wit , and J. Havenhand . 2024. High Temporal Resolution Environmental Data From Eelgrass Meadow [Dataset]. University of Gothenburg. 10.5878/KWXF-D021.

[eva70127-bib-0051] Jahnke, M. , and P. R. Jonsson . 2022. “Biophysical Models of Dispersal Contribute to Seascape Genetic Analyses.” Philosophical Transactions of the Royal Society of London. Series B, Biological Sciences 377, no. 1846: 20210024. 35067094 10.1098/rstb.2021.0024 PMC8784932

[eva70127-bib-0052] Jahnke, M. , P. R. Jonsson , P.‐O. Moksnes , L.‐O. Loo , M. Nilsson Jacobi , and J. L. Olsen . 2018. “Seascape Genetics and Biophysical Connectivity Modelling Support Conservation of the Seagrass *Zostera marina* in the Skagerrak‐Kattegat Region of the Eastern North Sea.” Evolutionary Applications 11, no. 5: 645–661. 29875808 10.1111/eva.12589 PMC5979629

[eva70127-bib-0053] Jahnke, M. , P.‐O. Moksnes , J. L. Olsen , et al. 2020. “Integrating Genetics, Biophysical, and Demographic Insights Identifies Critical Sites for Seagrass Conservation.” Ecological Applications: A Publication of the Ecological Society of America 30, no. 6: e02121. 32159897 10.1002/eap.2121

[eva70127-bib-0054] Jahnke, M. , I. A. Serra , G. Bernard , and G. Procaccini . 2015. “The Importance of Genetic Make‐Up in Seagrass Restoration: A Case Study of the Seagrass *Zostera noltei* .” Marine Ecology Progress Series 532: 111–122.

[eva70127-bib-0055] Jakobsson‐Thor, S. , J. Brakel , G. B. Toth , and H. Pavia . 2020. “Complex Interactions of Temperature, Light and Tissue Damage on Seagrass Wasting Disease in *Zostera marina* .” Frontiers in Marine Science 7: 575183.

[eva70127-bib-0056] Jeffery, N. , B. Vercaemer , R. Stanley , T. Kess , F. Dufresne , and M. Wong . 2023. “Variation in Genomic Vulnerability to Climate Change Across Temperate Populations of Eelgrass (*Zostera marina*).” Authorea Preprints. 10.22541/au.167291318.80887192/v3. PMC11033490 38650965

[eva70127-bib-0057] Jombart, T. , and I. Ahmed . 2011. “Adegenet 1.3‐1: New Tools for the Analysis of Genome‐Wide SNP Data.” Bioinformatics 27, no. 21: 3070–3071. 21926124 10.1093/bioinformatics/btr521 PMC3198581

[eva70127-bib-0058] Jones, T. A. 2003. “The Restoration Gene Pool Concept: Beyond the Native Versus Non‐Native Debate.” Restoration Ecology 11, no. 3: 281–290.

[eva70127-bib-0059] Jueterbock, A. , B. Duarte , J. Coyer , et al. 2021. “Adaptation of Temperate Seagrass to Arctic Light Relies on Seasonal Acclimatization of Carbon Capture and Metabolism.” Frontiers in Plant Science 12: 745855. 34925400 10.3389/fpls.2021.745855 PMC8675887

[eva70127-bib-0060] Jueterbock, A. , S. U. Franssen , N. Bergmann , et al. 2016. “Phylogeographic Differentiation Versus Transcriptomic Adaptation to Warm Temperatures in *Zostera marina* , a Globally Important Seagrass.” Molecular Ecology 25, no. 21: 5396–5411. 27598849 10.1111/mec.13829

[eva70127-bib-0061] Jump, A. S. , R. Marchant , and J. Peñuelas . 2009. “Environmental Change and the Option Value of Genetic Diversity.” Trends in Plant Science 14, no. 1: 51–58. 19042147 10.1016/j.tplants.2008.10.002

[eva70127-bib-0062] Kamvar, Z. N. , J. F. Tabima , and N. J. Grünwald . 2014. “Poppr: An R Package for Genetic Analysis of Populations With Clonal, Partially Clonal, and/or Sexual Reproduction.” PeerJ 2: e281. 24688859 10.7717/peerj.281 PMC3961149

[eva70127-bib-0063] Kettenring, K. M. , K. L. Mercer , C. Reinhardt Adams , and J. Hines . 2014. “EDITOR'S CHOICE: Application of Genetic Diversity–Ecosystem Function Research to Ecological Restoration.” Journal of Applied Ecology 51, no. 2: 339–348.

[eva70127-bib-0064] Langmead, B. , and S. L. Salzberg . 2012. “Fast Gapped‐Read Alignment With Bowtie 2.” Nature Methods 9, no. 4: 357–359. 22388286 10.1038/nmeth.1923 PMC3322381

[eva70127-bib-0065] Legendre, P. , and M. De Cáceres . 2013. “Beta Diversity as the Variance of Community Data: Dissimilarity Coefficients and Partitioning.” Ecology Letters 16, no. 8: 951–963. 23809147 10.1111/ele.12141

[eva70127-bib-0066] Legendre, P. , and L. Legendre . 2012. “Canonical Analysis.” In Developments in Environmental Modelling, edited by P. Legendre and L. Legendre , vol. 24, 625–710. Elsevier.

[eva70127-bib-0067] Legendre, P. , J. Oksanen , and C. J. F. ter Braak . 2011. “Testing the Significance of Canonical Axes in Redundancy Analysis: Test of Canonical Axes in RDA.” Methods in Ecology and Evolution 2, no. 3: 269–277.

[eva70127-bib-0068] Liggins, L. , E. A. Treml , and C. Riginos . 2019. “Seascape Genomics: Contextualizing Adaptive and Neutral Genomic Variation in the Ocean Environment.” In Population, Genomics, 171–218. Springer International Publishing.

[eva70127-bib-0069] Luu, K. , E. Bazin , and M. G. B. Blum . 2017. “Pcadapt: An R Package to Perform Genome Scans for Selection Based on Principal Component Analysis.” Molecular Ecology Resources 17, no. 1: 67–77. 27601374 10.1111/1755-0998.12592

[eva70127-bib-0070] Ma, X. , J. L. Olsen , T. B. H. Reusch , et al. 2021. “Improved Chromosome‐Level Genome Assembly and Annotation of the Seagrass, *Zostera marina* (Eelgrass).” F1000Research 10: 289. 34621505 10.12688/f1000research.38156.1 PMC8482049

[eva70127-bib-0071] Matz, M. V. 2021. “2bRAD_denovo: Genome‐Wide De Novo Genotyping With 2bRAD.” https://github.com/z0on/2bRAD_denovo.

[eva70127-bib-0072] Meirmans, P. G. 2024. “Correcting for Replicated Genotypes May Introduce More Problems Than It Solves.” Molecular Ecology Resources 25: e14041. 39465502 10.1111/1755-0998.14041 PMC11887605

[eva70127-bib-0073] Meysick, L. , E. Infantes , L. Rugiu , K. Gagnon , and C. Boström . 2022. “Coastal Ecosystem Engineers and Their Impact on Sediment Dynamics: Eelgrass–Bivalve Interactions Under Wave Exposure.” Limnology and Oceanography 67, no. 3: 621–633.

[eva70127-bib-0074] Moksnes, P.‐O. , and P. Bergström . 2025. *Analys av ålgräsets Historiska Areella Utbredning i Västerhavet: Metoder, Statusbedömning Och Analys av Möjliga Orsaker Till Förändringar* . Swedish Agency for Marine and Water Management.

[eva70127-bib-0075] Moksnes, P.‐O. , L. Gipperth , L. Eriander , K. Laas , S. Cole , and E. Infantes . 2016. Förvaltning Och Restaurering av Ålgräs i Sverige: Ekologisk, Juridisk Och Ekonomisk Bakgrund. Digitala Vetenskapliga Arkivet, Swedish Agency for Marine and Water Management (Havs‐Och Vattenmyndigheten). https://urn.kb.se/resolve?urn=urn:nbn:se:havochvatten:diva‐107.

[eva70127-bib-0076] Moksnes, P.‐O. , M. Gullström , K. Tryman , and S. Baden . 2008. “Trophic Cascades in a Temperate Seagrass Community.” Oikos 117, no. 5: 763–777.

[eva70127-bib-0077] Moksnes, P.‐O. , M. E. Röhr , M. Holmer , et al. 2021. “Major Impacts and Societal Costs of Seagrass Loss on Sediment Carbon and Nitrogen Stocks.” Ecosphere 12, no. 7: e03658. 10.1002/ecs2.3658.

[eva70127-bib-0078] Nadeau, C. P. , A. R. Hughes , E. G. Schneider , P. Colarusso , N. A. Fisichelli , and A. J. Miller‐Rushing . 2024. “Incorporating Experiments Into Management to Facilitate Rapid Learning About Climate Change Adaptation.” Biological Conservation 289: 110374.

[eva70127-bib-0079] Nakagawa, S. , D. W. A. Noble , M. Lagisz , R. Spake , W. Viechtbauer , and A. M. Senior . 2023. “A Robust and Readily Implementable Method for the Meta‐Analysis of Response Ratios With and Without Missing Standard Deviations.” Ecology Letters 26, no. 2: 232–244. 36573275 10.1111/ele.14144 PMC10108319

[eva70127-bib-0080] Nielsen, E. S. , M. Beger , R. Henriques , and S. von der Heyden . 2023. “Integrating Environmental, Evolutionary, and Socioeconomic Vulnerability to Future‐Proof Coastal Conservation Planning.” Biological Conservation 286: 110302.

[eva70127-bib-0081] Nielsen, E. S. , J. O. Hanson , S. B. Carvalho , et al. 2023. “Molecular Ecology Meets Systematic Conservation Planning.” Trends in Ecology & Evolution 38, no. 2: 143–155. 36210287 10.1016/j.tree.2022.09.006

[eva70127-bib-0082] Nimbs, M. J. , T. M. Glasby , E. A. Sinclair , D. Swadling , T. R. Davis , and M. A. Coleman . 2024. “A Donor Registry: Genomic Analyses of *Posidonia australis* Seagrass Meadows Identifies Adaptive Genotypes for Future‐Proofing.” Ecology and Evolution 14, no. 12: e70667. 39650543 10.1002/ece3.70667 PMC11622155

[eva70127-bib-0083] Nordlund, L. M. , R. K. F. Unsworth , S. Wallner‐Hahn , et al. 2024. “One Hundred Priority Questions for Advancing Seagrass Conservation in Europe.” Plants, People, Planet 6: 587–603. 10.1002/ppp3.10486.

[eva70127-bib-0084] Obenchain, V. , M. Lawrence , V. Carey , S. Gogarten , P. Shannon , and M. Morgan . 2014. “VariantAnnotation: A Bioconductor Package for Exploration and Annotation of Genetic Variants.” Bioinformatics (Oxford, England) 30, no. 14: 2076–2078. 24681907 10.1093/bioinformatics/btu168 PMC4080743

[eva70127-bib-0085] Oksanen, J. , G. L. Simpson , F. G. Blanchet , et al. 2024. “vegan: Community Ecology Package.” https://vegandevs.github.io/vegan/.

[eva70127-bib-0086] Olsen, J. L. , J. A. Coyer , and B. Chesney . 2014. “Numerous Mitigation Transplants of the Eelgrass *Zostera marina* in Southern California Shuffle Genetic Diversity and May Promote Hybridization With *Zostera pacifica* .” Biological Conservation 176: 133–143.

[eva70127-bib-0087] Orth, R. J. , K. A. Moore , S. R. Marion , D. J. Wilcox , and D. B. Parrish . 2012. “Seed Addition Facilitates Eelgrass Recovery in a Coastal Bay System.” Marine Ecology Progress Series 448: 177–195.

[eva70127-bib-0088] Paradis, E. , and K. Schliep . 2019. “Ape 5.0: An Environment for Modern Phylogenetics and Evolutionary Analyses in R.” Bioinformatics (Oxford, England) 35, no. 3: 526–528. 30016406 10.1093/bioinformatics/bty633

[eva70127-bib-0089] Pazzaglia, J. , H. M. Nguyen , A. Santillán‐Sarmiento , et al. 2021. “The Genetic Component of Seagrass Restoration: What We Know and the Way Forwards.” Watermark 13, no. 6: 829.

[eva70127-bib-0090] Peres‐Neto, P. R. , P. Legendre , S. Dray , and D. Borcard . 2006. “Variation Partitioning of Species Data Matrices: Estimation and Comparison of Fractions.” Ecology 87, no. 10: 2614–2625. 17089669 10.1890/0012-9658(2006)87[2614:vposdm]2.0.co;2

[eva70127-bib-0091] Pereyra, R. T. , A. Kinnby , A. Le Moan , et al. 2025. “An Evolutionary Mosaic Challenges Traditional Monitoring of a Foundation Species in a Coastal Environment‐The Baltic *Fucus vesiculosus* .” Molecular Ecology: e17699. 39957665 10.1111/mec.17699 PMC12684334

[eva70127-bib-0092] Posit team . 2024. “RStudio: Integrated Development Environment for R.” Posit Software, PBC. http://www.posit.co/.

[eva70127-bib-0093] Procaccini, G. , and L. Piazzi . 2001. “Genetic Polymorphism and Transplantation Success in the Mediterranean Seagrass *Posidonia oceanica* .” Restoration Ecology 9, no. 3: 332–338.

[eva70127-bib-0094] R Core Team . 2024. R: A Language and Environment for Statistical Computing. R Foundation for Statistical Computing. https://www.R‐project.org/.

[eva70127-bib-0095] Rasmussen, E. 1977. “The Wasting Disease of Eelgrass (*Zostera marina*) and Its Effects on Environmental Factors and Fauna.” Seagrass Ecosystems: 1–51.

[eva70127-bib-0096] Reusch, T. B. H. , I. B. Baums , and B. Werner . 2021. “Evolution via Somatic Genetic Variation in Modular Species.” Trends in Ecology & Evolution 36, no. 12: 1083–1092. 34538501 10.1016/j.tree.2021.08.011

[eva70127-bib-0097] Reusch, T. B. H. , A. Ehlers , A. Hämmerli , and B. Worm . 2005. “Ecosystem Recovery After Climatic Extremes Enhanced by Genotypic Diversity.” Proceedings of the National Academy of Sciences of the United States of America 102, no. 8: 2826–2831. 15710890 10.1073/pnas.0500008102 PMC549506

[eva70127-bib-0098] Reynolds, L. K. , K. J. McGlathery , and M. Waycott . 2012. “Genetic Diversity Enhances Restoration Success by Augmenting Ecosystem Services.” PLoS One 7, no. 6: e38397. 22761681 10.1371/journal.pone.0038397 PMC3382623

[eva70127-bib-0099] Reynolds, L. K. , M. Waycott , K. J. McGlathery , R. J. Orth , and J. C. Zieman . 2012. “Eelgrass Restoration by Seed Maintains Genetic Diversity: Case Study From a Coastal Bay System.” Marine Ecology Progress Series 448: 223–233.

[eva70127-bib-0100] Ries, S. R. , E. Faust , K. Johannesson , et al. 2023. “Genetic Structure and Diversity of the Seagrass *Zostera marina* Along a Steep Environmental Gradient, With Implications for Genetic Monitoring.” Frontiers in Climate 5: 1303337. 10.3389/fclim.2023.1303337.

[eva70127-bib-0102] Rossetto, M. , J. Bragg , A. Kilian , H. McPherson , M. van der Merwe , and P. D. Wilson . 2019. “Restore and Renew: A Genomics‐Era Framework for Species Provenance Delimitation: Species Provenance Delimitation Framework.” Restoration Ecology 27, no. 3: 538–548.

[eva70127-bib-0103] Ruocco, M. , M. Jahnke , J. Silva , G. Procaccini , and E. Dattolo . 2022. “2b‐RAD Genotyping of the Seagrass *Cymodocea nodosa* Along a Latitudinal Cline Identifies Candidate Genes for Environmental Adaptation.” Frontiers in Genetics 13: 866758. 10.3389/fgene.2022.866758. 35651946 PMC9149362

[eva70127-bib-0104] Schiebelhut, L. M. , R. K. Grosberg , J. J. Stachowicz , and R. A. Bay . 2023. “Genomic Responses to Parallel Temperature Gradients in the Eelgrass *Zostera marina* in Adjacent Bays.” Molecular Ecology 32, no. 11: 2835–2849. 36814144 10.1111/mec.16899

[eva70127-bib-0105] Sinclair, E. A. , J. Verduin , S. L. Krauss , J. Hardinge , J. Anthony , and G. A. Kendrick . 2013. “A Genetic Assessment of a Successful Seagrass Meadow (*Posidonia australis*) Restoration Trial.” Ecological Management & Restoration 14, no. 1: 68–71.

[eva70127-bib-0106] Sotka, E. E. , A. R. Hughes , T. C. Hanley , and C. G. Hays . 2024. “Restricted Dispersal and Phenotypic Response to Water Depth in a Foundation Seagrass.” Molecular Ecology 33, no. 23: e17565. 39474794 10.1111/mec.17565 PMC11589694

[eva70127-bib-0107] Tomas, F. , J. M. Abbott , C. Steinberg , M. Balk , S. L. Williams , and J. J. Stachowicz . 2011. “Plant Genotype and Nitrogen Loading Influence Seagrass Productivity, Biochemistry, and Plant‐Herbivore Interactions.” Ecology 92, no. 9: 1807–1817. 21939077 10.1890/10-2095.1

[eva70127-bib-0108] Torn, K. , A. Peterson , and K. Herkül . 2020. “Predicting the Impact of Climate Change on the Distribution of the Key Habitat‐Forming Species in the ne Baltic Sea.” Journal of Coastal Research 95: 177.

[eva70127-bib-0109] Unsworth, R. K. F. , C. J. Collier , G. M. Henderson , and L. J. McKenzie . 2012. “Tropical Seagrass Meadows Modify Seawater Carbon Chemistry: Implications for Coral Reefs Impacted by Ocean Acidification.” Environmental Research Letters 7, no. 2: 024026.

[eva70127-bib-0110] van Buuren, S. , and K. Groothuis‐Oudshoorn . 2011. “Mice: Multivariate Imputation by Chained Equations in R.” Journal of Statistical Software 45, no. 3: 1–67.

[eva70127-bib-0111] van Katwijk, M. M. , A. R. Bos , V. N. de Jonge , L. S. A. M. Hanssen , D. C. R. Hermus , and D. J. de Jong . 2009. “Guidelines for Seagrass Restoration: Importance of Habitat Selection and Donor Population, Spreading of Risks, and Ecosystem Engineering Effects.” Marine Pollution Bulletin 58, no. 2: 179–188. 19131078 10.1016/j.marpolbul.2008.09.028

[eva70127-bib-0112] van Katwijk, M. M. , G. H. W. Schmitz , L. S. Hanssen , and C. den Hartog . 1998. “Suitability of *Zostera marina* Populations for Transplantation to the Wadden Sea as Determined by a Mesocosm Shading Experiment.” Aquatic Botany 60, no. 4: 283–305.

[eva70127-bib-0113] van Katwijk, M. M. , A. Thorhaug , N. Marbà , et al. 2016. “Global Analysis of Seagrass Restoration: The Importance of Large‐Scale Planting.” Journal of Applied Ecology 53, no. 2: 567–578.

[eva70127-bib-0114] van Katwijk, M. M. , B. I. van Tussenbroek , S. Hanssen , A. Hendriks , and L. Hanssen . 2021. “Rewilding the Sea With Domesticated Seagrass.” Bioscience 71, no. 11: 1171–1178. 34733118 10.1093/biosci/biab092 PMC8560307

[eva70127-bib-0115] van Oppen, M. J. H. , and M. A. Coleman . 2022. “Advancing the Protection of Marine Life Through Genomics.” PLoS Biology 20, no. 10: e3001801. 36251637 10.1371/journal.pbio.3001801 PMC9576104

[eva70127-bib-0116] van Oppen, M. J. H. , R. D. Gates , L. L. Blackall , et al. 2017. “Shifting Paradigms in Restoration of the World's Coral Reefs.” Global Change Biology 23, no. 9: 3437–3448. 28247459 10.1111/gcb.13647

[eva70127-bib-0117] Wang, S. , E. Meyer , J. K. McKay , and M. V. Matz . 2012. “2b‐RAD: A Simple and Flexible Method for Genome‐Wide Genotyping.” Nature Methods 9, no. 8: 808–810. 22609625 10.1038/nmeth.2023

[eva70127-bib-0118] Waycott, M. , C. M. Duarte , T. J. B. Carruthers , et al. 2009. “Accelerating Loss of Seagrasses Across the Globe Threatens Coastal Ecosystems.” Proceedings of the National Academy of Sciences of the United States of America 106, no. 30: 12377–12381. 19587236 10.1073/pnas.0905620106 PMC2707273

[eva70127-bib-0119] Weeks, A. R. , C. M. Sgro , A. G. Young , et al. 2011. “Assessing the Benefits and Risks of Translocations in Changing Environments: A Genetic Perspective: Translocations in Changing Environments.” Evolutionary Applications 4, no. 6: 709–725. 22287981 10.1111/j.1752-4571.2011.00192.x PMC3265713

[eva70127-bib-0120] Wickham, H. 2016. ggplot2: Elegant Graphics for Data Analysis. Springer‐Verlag New York. https://ggplot2.tidyverse.org.

[eva70127-bib-0121] Wood, G. , E. M. Marzinelli , A. H. Campbell , P. D. Steinberg , A. Vergés , and M. A. Coleman . 2021. “Genomic Vulnerability of a Dominant Seaweed Points to Future‐Proofing Pathways for Australia's Underwater Forests.” Global Change Biology 27, no. 10: 2200–2212. 33511779 10.1111/gcb.15534

[eva70127-bib-0122] Wood, G. , E. M. Marzinelli , A. Vergés , A. H. Campbell , P. D. Steinberg , and M. A. Coleman . 2020. “Using Genomics to Design and Evaluate the Performance of Underwater Forest Restoration.” Journal of Applied Ecology 57, no. 10: 1988–1998.

[eva70127-bib-0123] Yu, L. , M. Khachaturyan , M. Matschiner , et al. 2023. “Ocean Current Patterns Drive the Worldwide Colonization of Eelgrass ( *Zostera marina* ).” Nature Plants 9: 1207–1220. 10.1038/s41477-023-01464-3. 37474781 PMC10435387

[eva70127-bib-0124] Yu, L. , J. Renton , A. Burian , et al. 2024. “A Somatic Genetic Clock for Clonal Species.” Nature Ecology & Evolution 8, no. 7: 1327–1336. 38858515 10.1038/s41559-024-02439-z PMC11239492

