# Supplementary material for: The Influence of Spatial Distance and Environment on Small‐Scale Genetic Variability in Eelgrass and Its Application for Restoration
Source: Evol Appl. 2025 Jul 11;18(7):e70127. doi: 10.1111/eva.70127 (PMC12246960; doi:10.1111/eva.70127)
Supplement: Supplementary file 1 — Data S1. [file EVA-18-e70127-s001.docx]

**Supplementary**

**Supplementary Figure 1.** Plotting of PCoA1 and PCoA2 of sea distance among the ten sampled eelgrass meadows as measured on google maps without crossing land. As expected, the eelgrass meadows plot according to their geographic location as shown in Fig. 1. Exposed sites are shown in blues, where increasing darkness indicates sites with decreasing TDiff (i.e. the site with the lowest TDiff is the darkest). Sheltered sites are shown in oranges, where increasing darkness indicates increasing TDiff (i.e. darkest colour is warmest site).


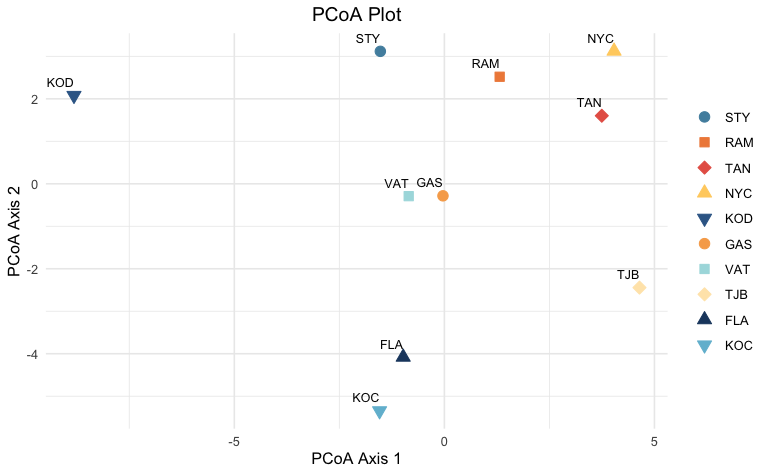


**Supplementary Figure 2.** Correlogram of all environmental variables available for the ten small-scale eelgrass meadows. Summarized by PC1-2 are the important components of genetic structure based on only intergenic loci, and PCoA1+2 are the components for geographic distance. Maximum and mean daily temperature showed correlations above the chosen threshold of 0.7 with each other as well as PCoA1, and Tmax with TDiff, and were therefore removed. Note however, that these variables are therefore still possible predictors of genetic variability. Left: correlation analysis for the clone corrected data set (111 MLLs and 124 samples). Right: correlation analysis for the dataset including all 189 samples.

*
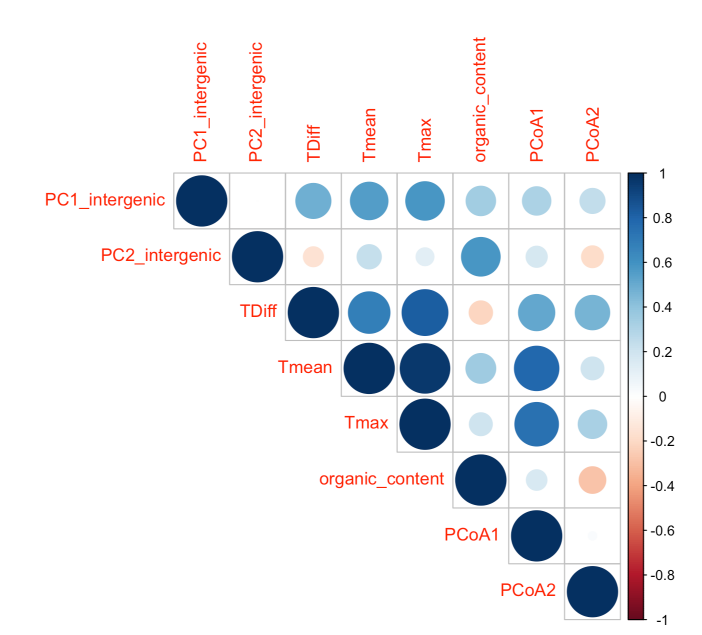
*
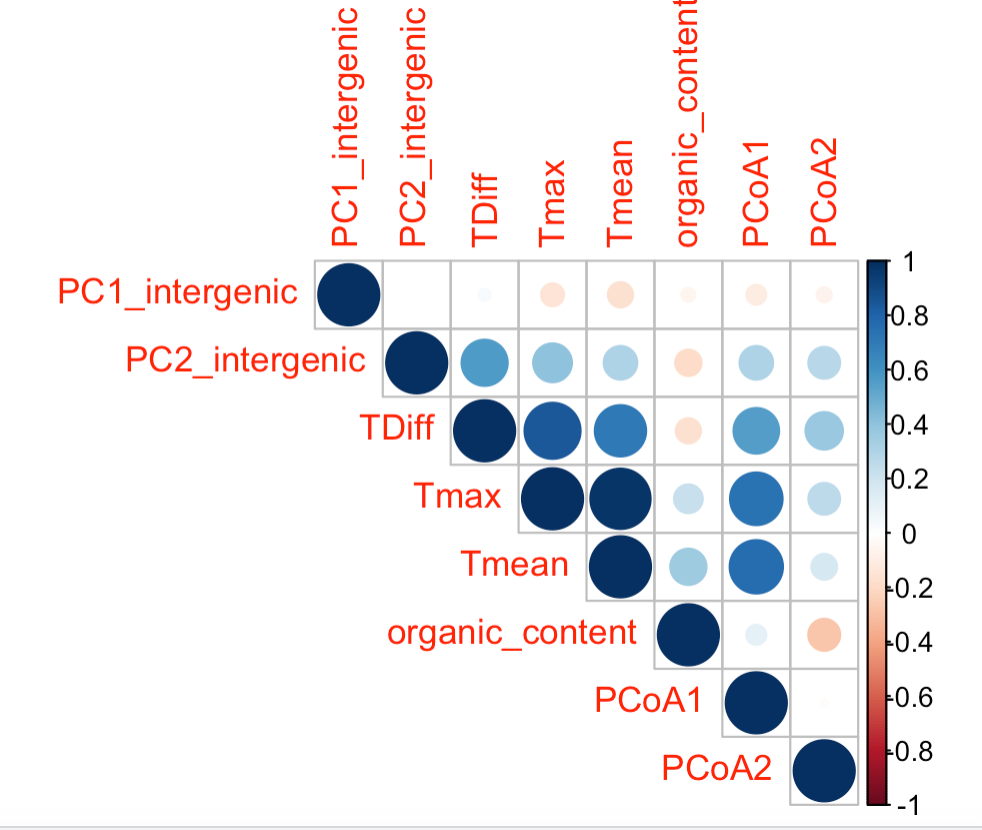


**Supplementary Figure 3.** PCA of all SNPs that are annotated as intergenic. To incorporate “neutral” population genetic structure as a predictor in the RDA, we first filtered the full SNP set to only maintain intergenic, i.e. more likely neutral, SNPs, which we located with “locateVariants()” from the package “VariantAnnotations 1.52.0” [(Obenchain et al., 2014)](https://paperpile.com/c/dOFlz0/IxSq). We then performed a PCA of the intergenic SNPs (total of 1,110 SNPs for the clone filtered dataset and 1,135 SNPs for the data set including all 189 samples) with the “rda” function, and selected the two first PCs axes, which explained a high proportion of genetic differentiation.

**
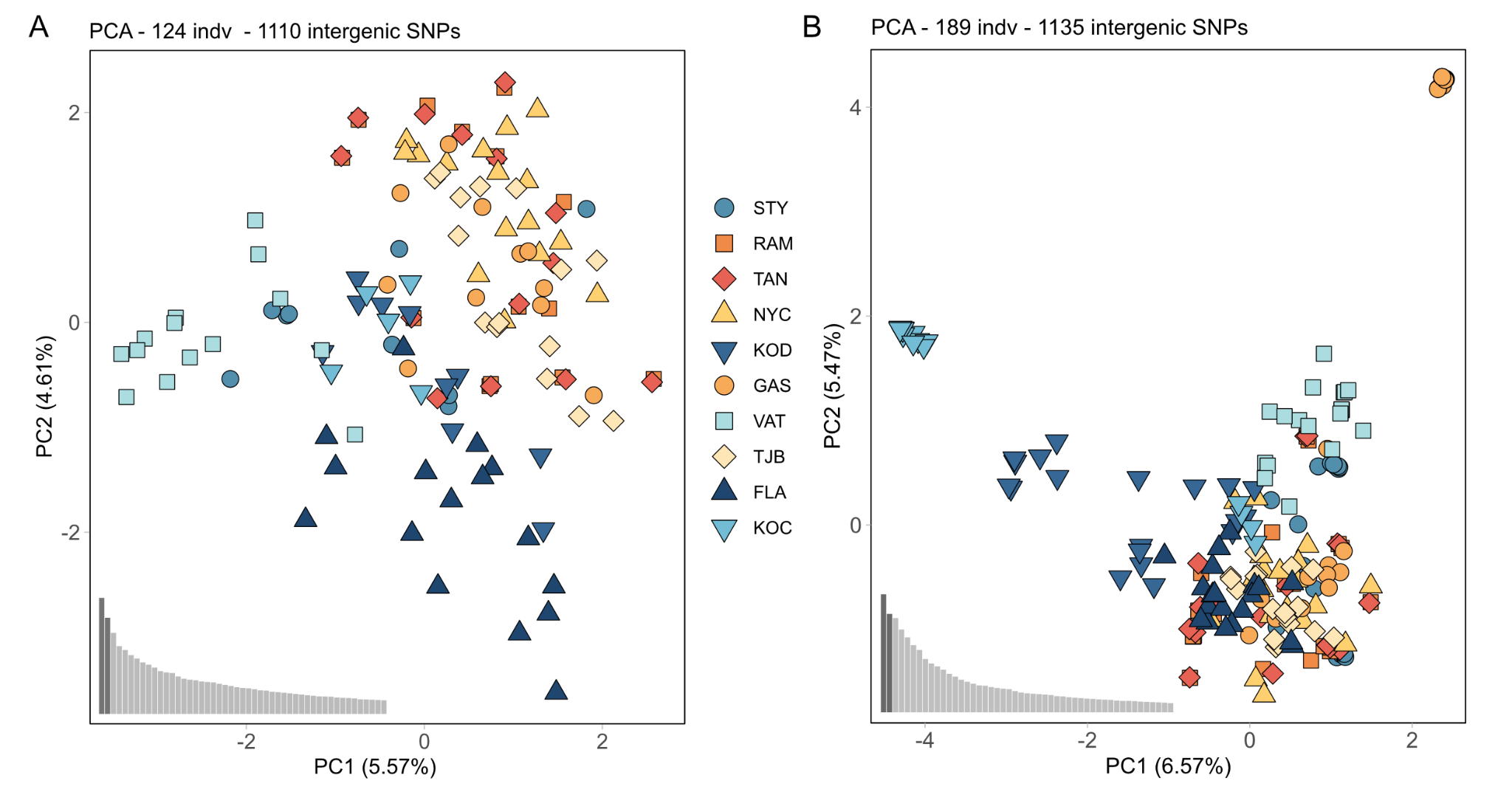
**

**Supplementary Figure 4.** Boxplots for total eelgrass growth across sites. The sites are ordered according to mean growth/site. Exposed sites are shown in blues, where increasing darkness indicates sites with decreasing TDiff (i.e. the site with the lowest TDiff is the darkest). Sheltered sites are shown in oranges, where increasing darkness indicates increasing TDiff (i.e. darkest orange indicates the warmest site). We also measured the emergence of new shoots, and sheltered meadows grew more shoots (mean: 19; range: 17-21 per site) than exposed meadows (mean: 15; range: 9-22 per site). The two exposed sites with the lowest mean individual growth, also had the lowest emergence of new shoots, and the three sheltered meadows with the highest mean growth, all had a high number of new shoots.

**
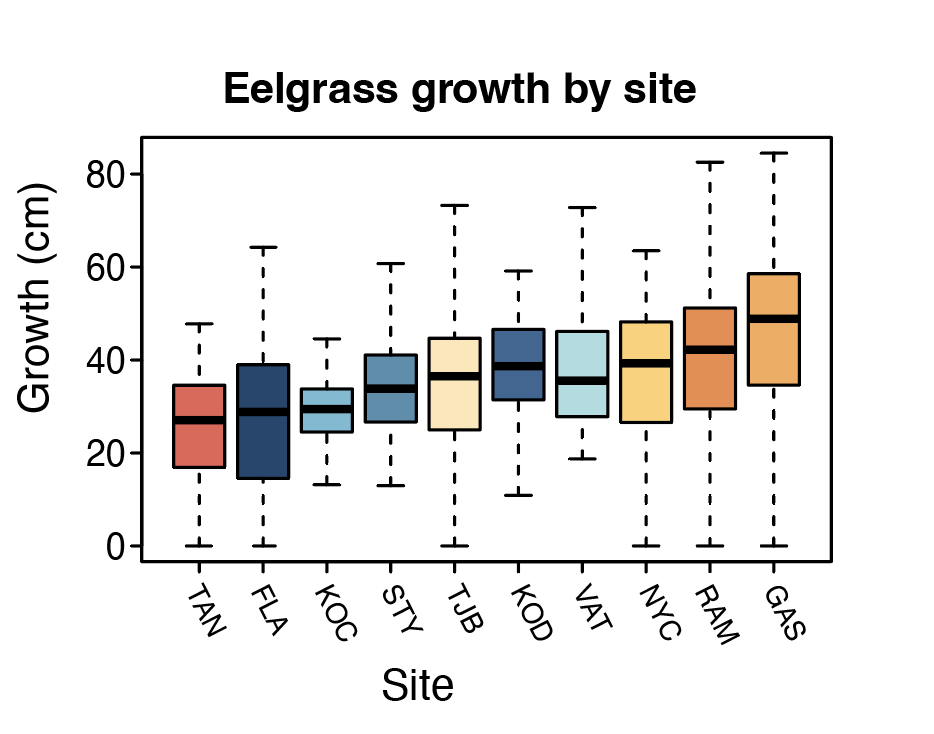
**

**Supplementary Figure 5.** Principal component analysis (PCA) based on all 189 samples genotyped with 1,689 SNPs from ten sampling sites along the Swedish west coast. Each dot represents one individual and the color/shape combination represents the sampling site. Warmer colors indicate sheltered and colder colors exposed sampling sites. The plot shows genetic differentiation for 189 samples along PCA axes 1 and 2, which were the most important according to Eigenvalues. Note that the inclusion of clones leads to clones clustering strongly together and away from unique MLLs. See Table 1 for acronyms.


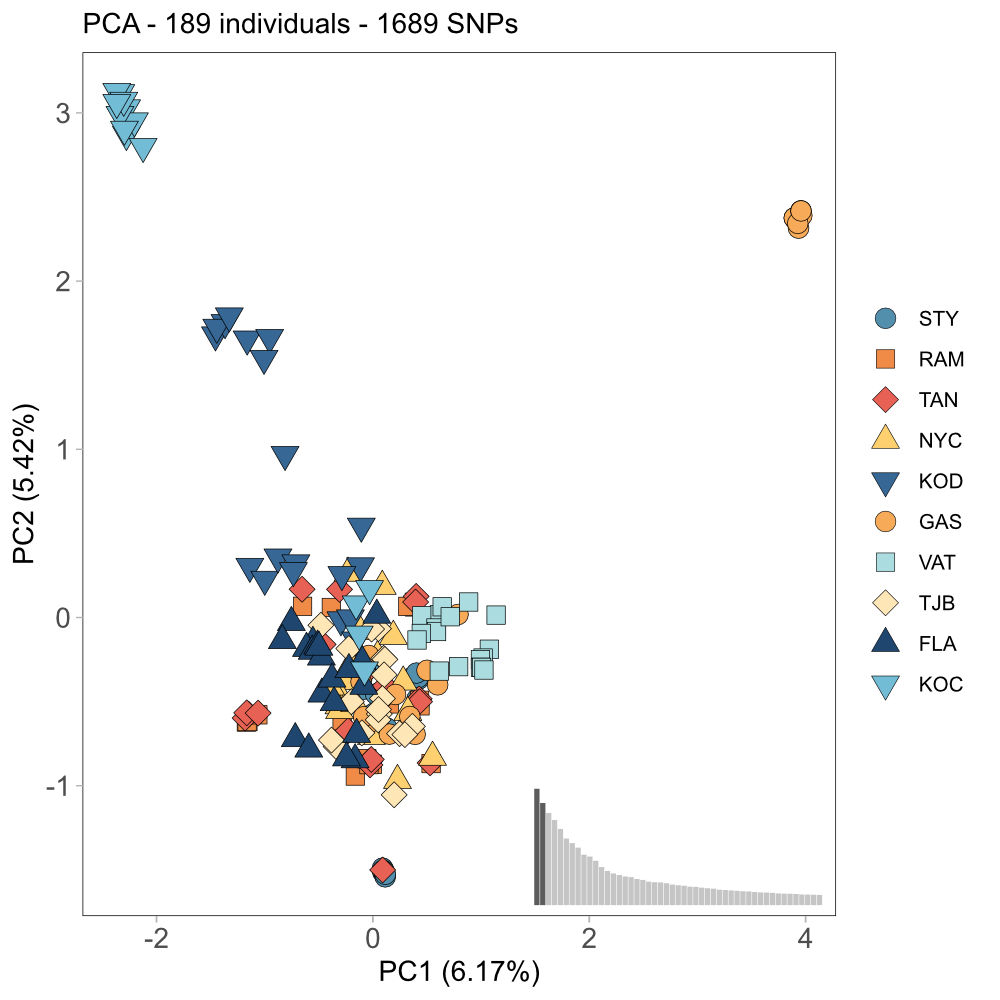


**Supplementary Figure 6.** Isolation-by-distance analysis using Mantel test to correlate sea distance (Supplementary Table 2) with pairwise Weir & Cockerham F_ST_ (Supplementary Table 3).


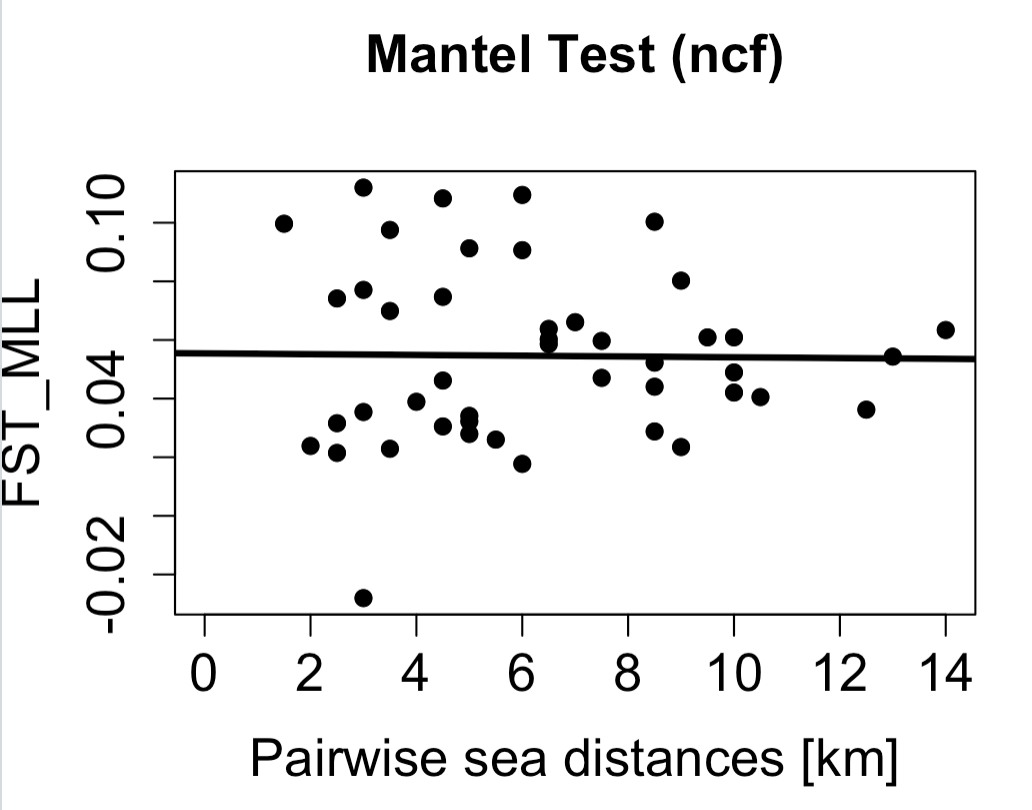


**Supplementary Figure 7.** RDA of the full model based on all polymorphic loci and all 189 eelgrass samples. Dots indicate individual genotypes, coloured according to the meadow of origin. Exposed sites are shown in blues, where increasing darkness indicates sites with decreasing TDiff (i.e. the site with the lowest TDiff is the darkest). Sheltered sites are shown in oranges, where increasing darkness indicates increasing TDiff (i.e. darkest colour is warmest site). Red arrows represent variables that drive the observed population structure.

**
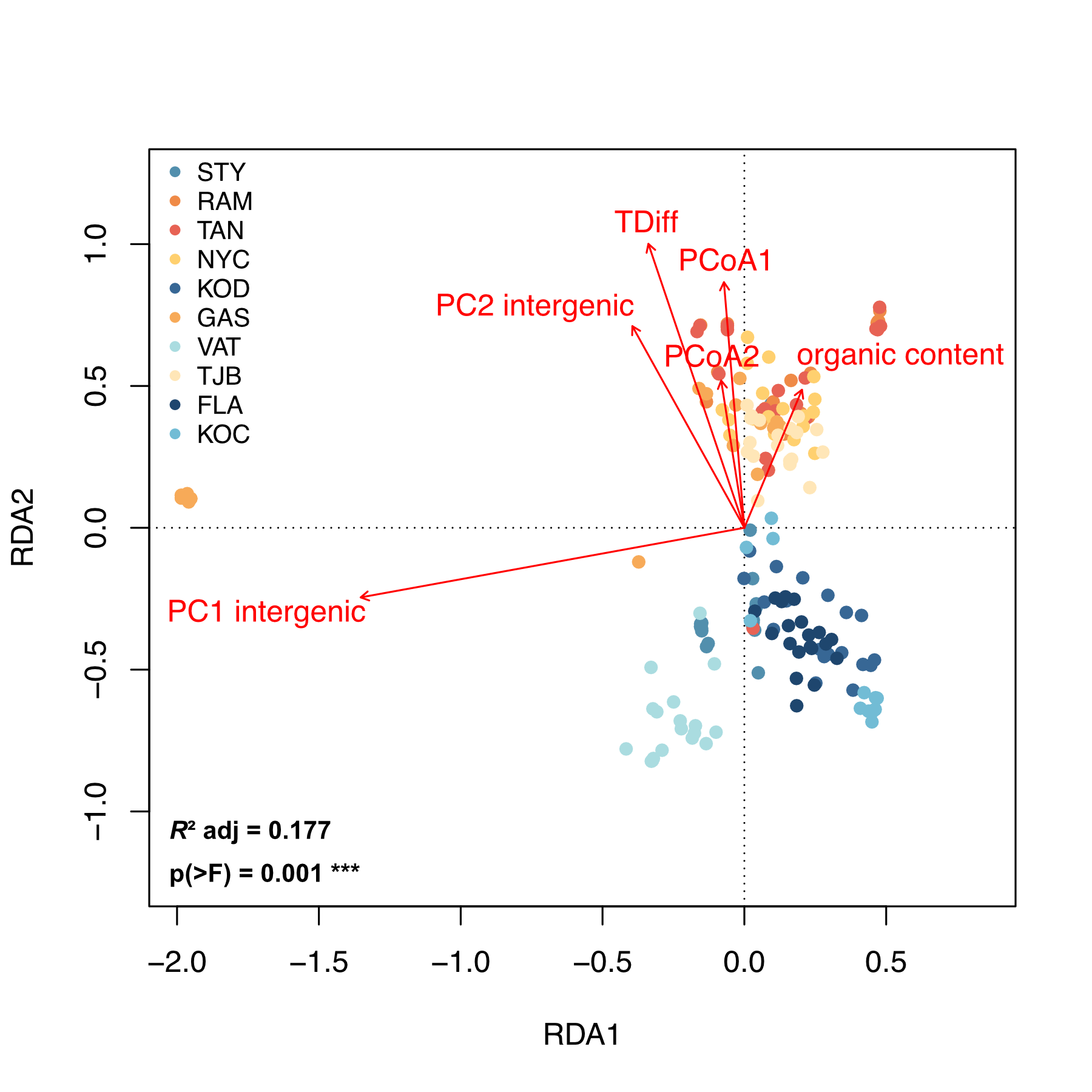
**

**Supplementary Figure 8** Detection of putative adaptive loci in the ten eelgrass meadows sampled along the Swedish coast. Left: Using RDAdapt we identified 112 putative outliers in our clone-corrected eelgrass dataset and genotyped with 1,648 SNPs, of which 36 can be considered “top outliers”, when setting the threshold for the p-value to 1e-21 based on visual inspection of the Manhattan plot. When basing the analysis on all 189 samples and 1,689 SNPs (shown on the right), we identified 218 putative outlier loci, of which 27 were “top outlier” loci, when setting the threshold to 1e-63 based on visual inspection of the Manhattan plot.


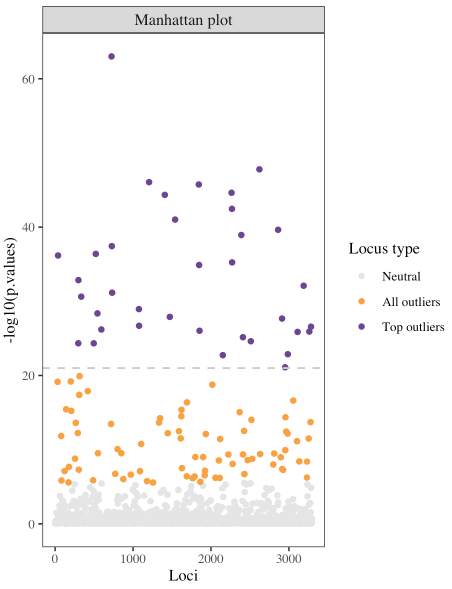

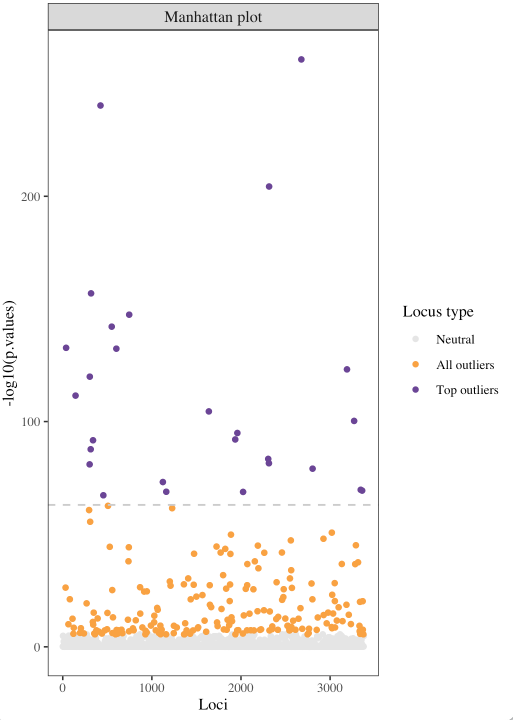


**Supplementary Figure 9.** Allele frequencies of the 22 outlier loci detected with both *pcadapt* and *rdadapt* in the clone corrected dataset. left) ordered from North to South, middle) ordered according to daily temperature variability (TDiff) and right) ordered according to organic content. See Table 1 for acronyms.


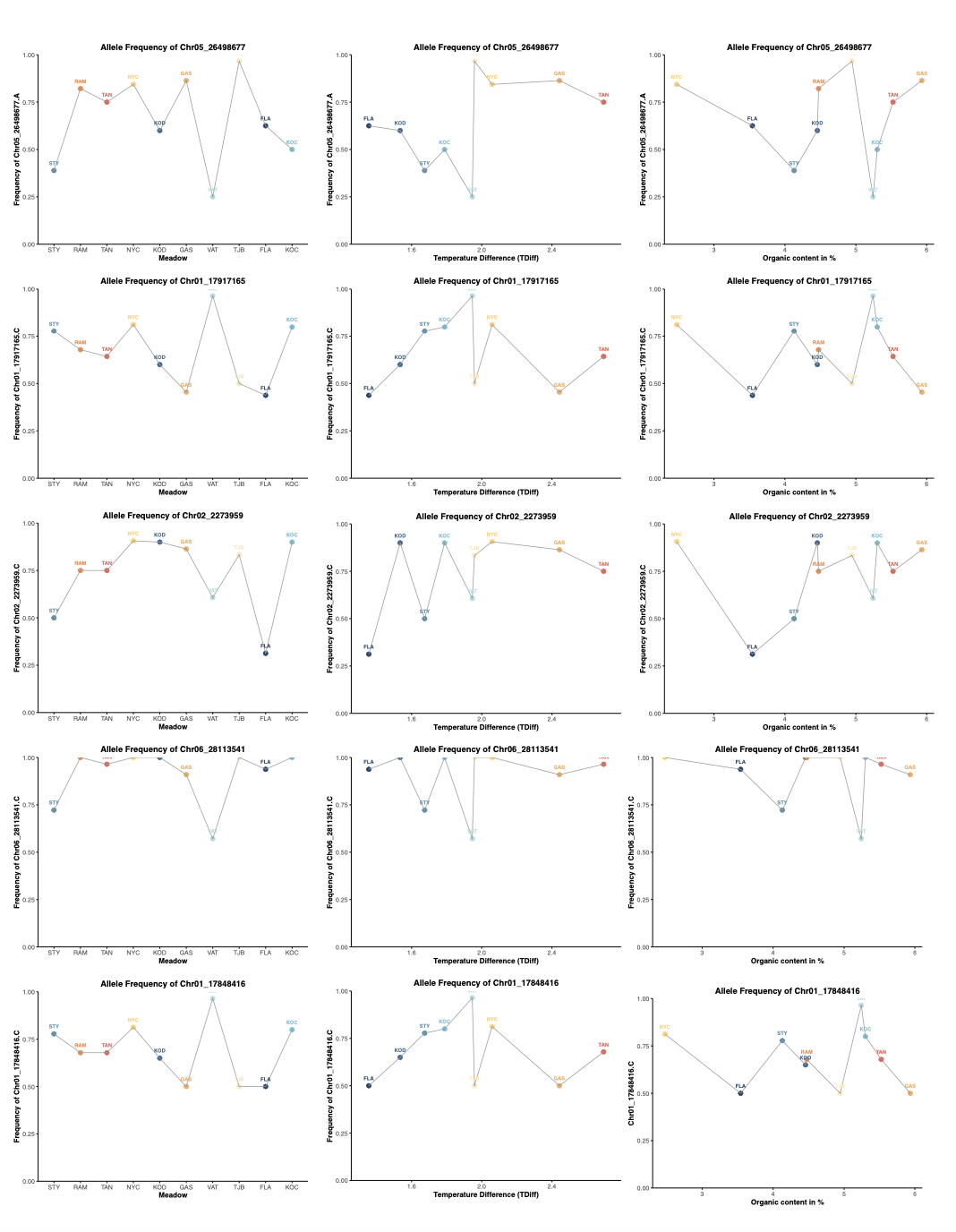


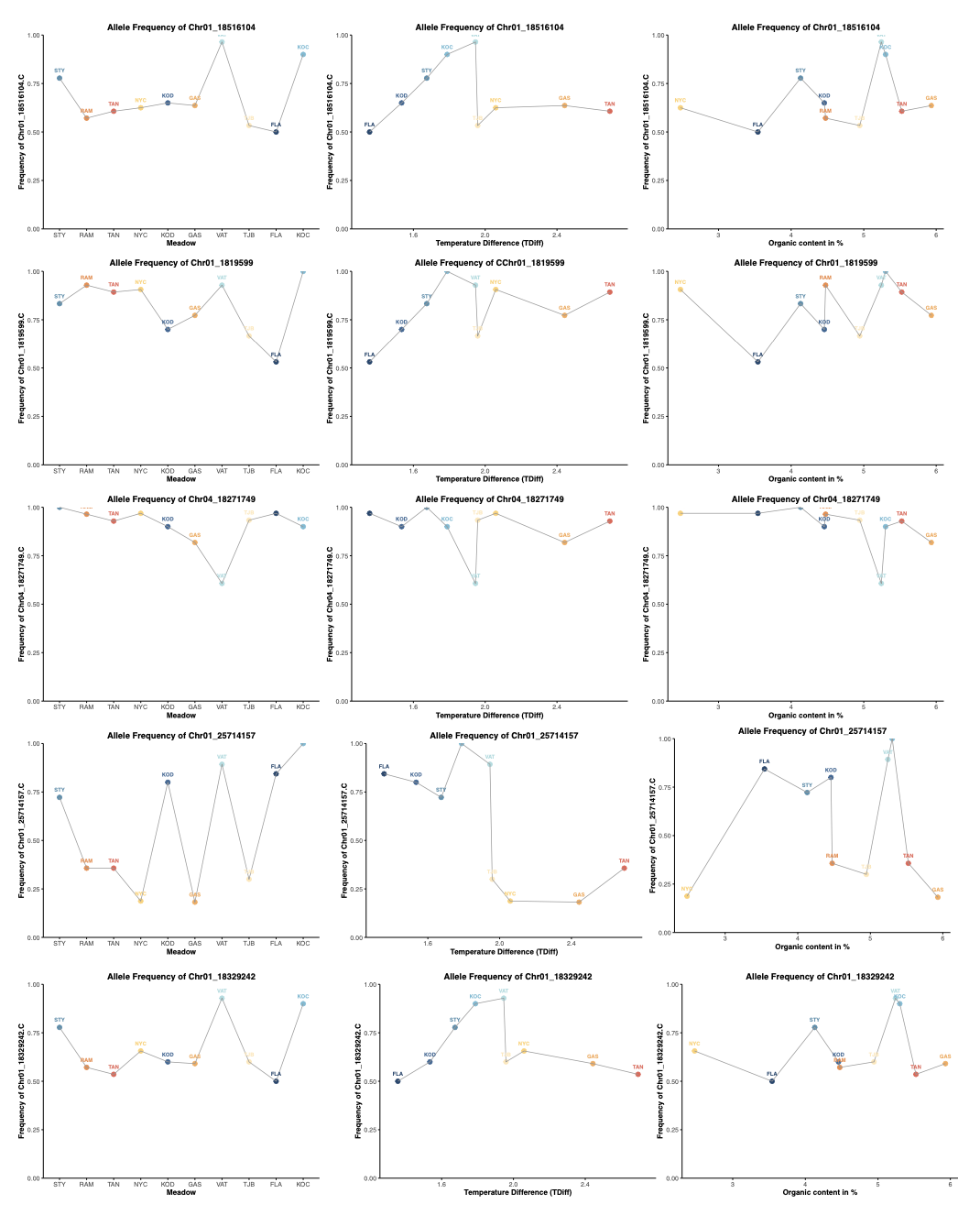


**
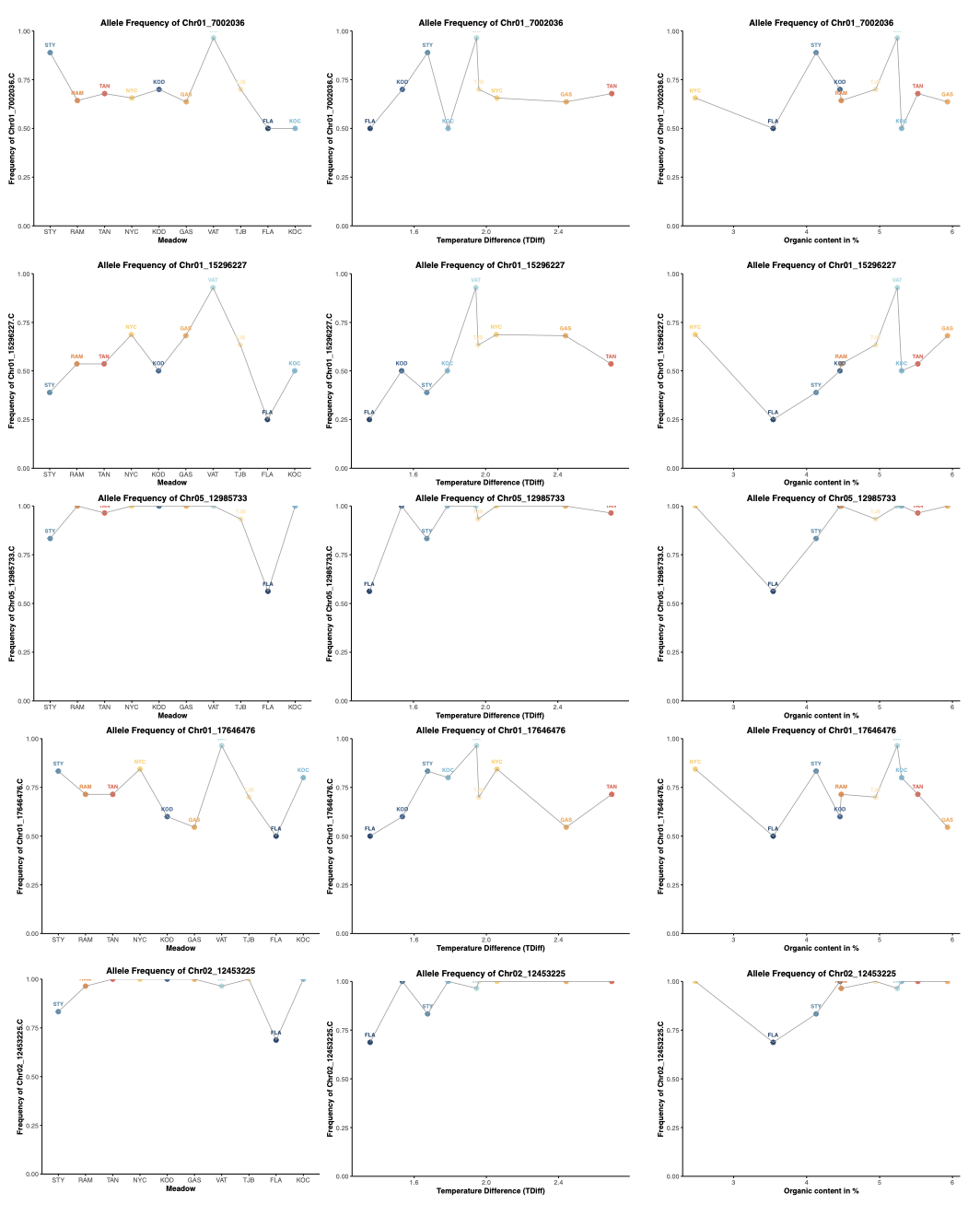
**

**
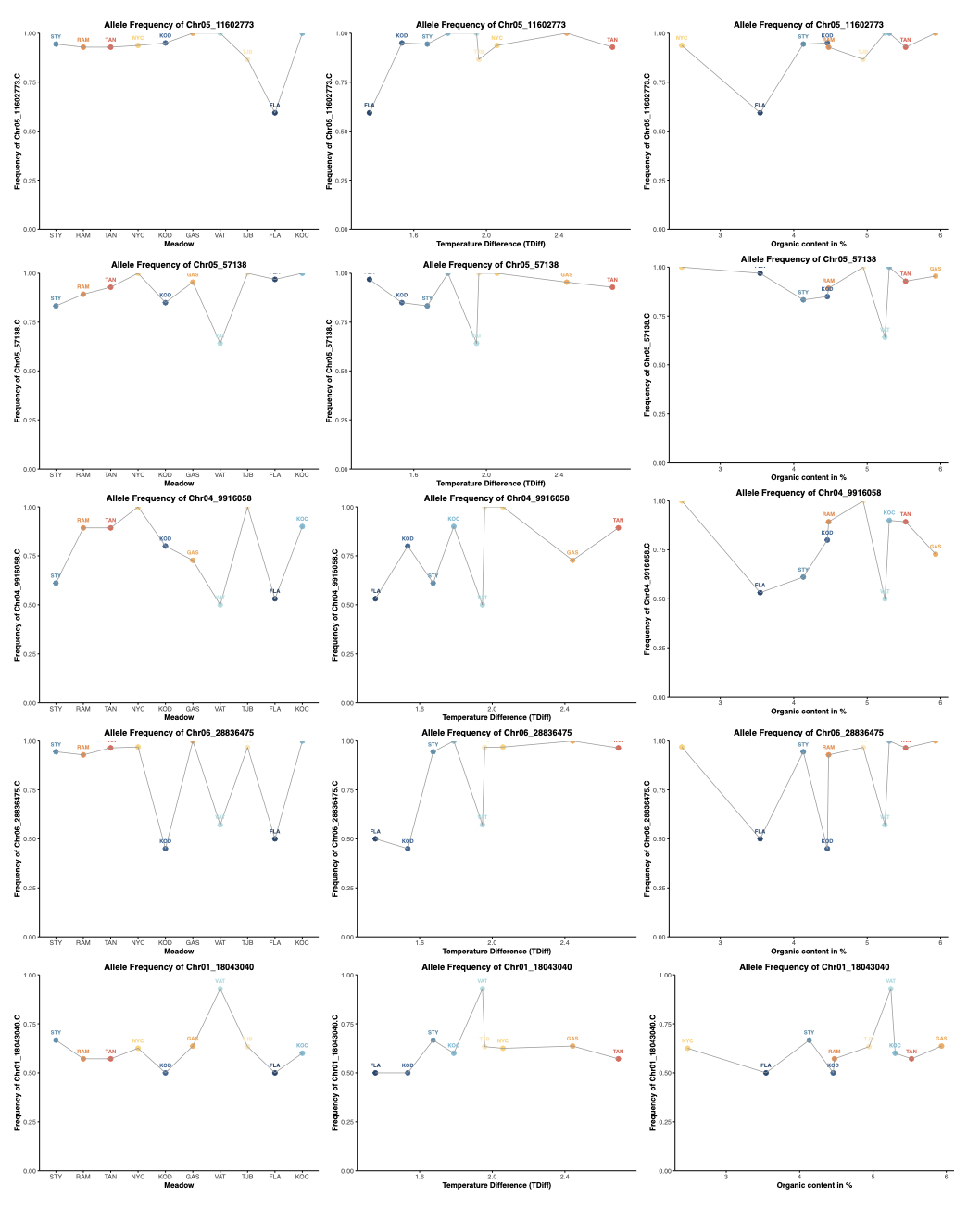
**

**
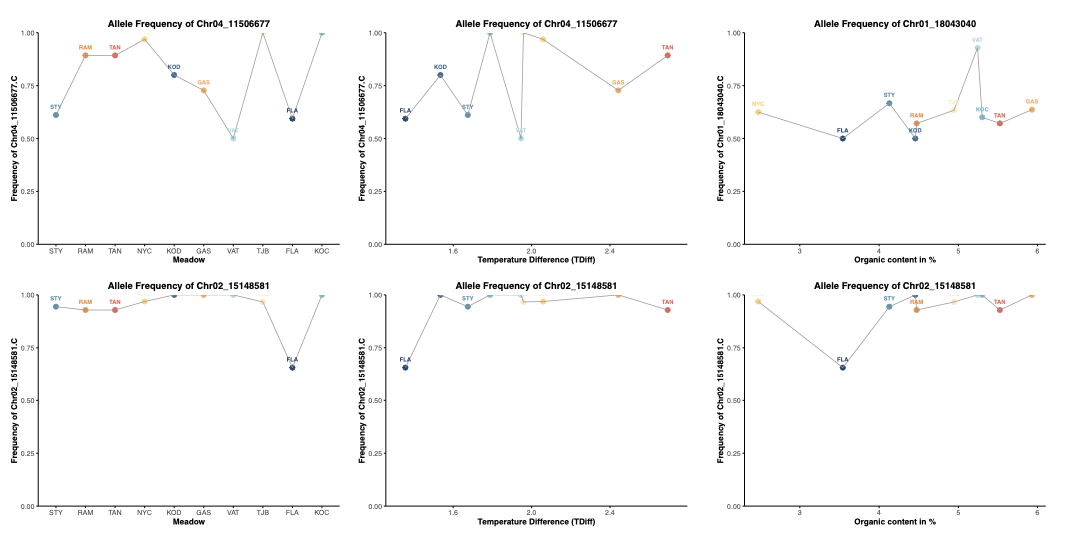
**

**Supplementary Figure 10.** a) Boxplot of total eelgrass growth across sites, extreme event treatment and provenance strategies (single- or mixed-meadow origin). A blue stripe on the x-axis indicates control, and a red stripe indicates the extreme event treatment with increased temperature and reduced salinity. b) Boxplots for total eelgrass growth across site and extreme event treatment. Exposed sites are shown in blues, where increasing darkness indicates sites with decreasing daily temperature range. Sheltered sites are shown in oranges, where increasing darkness indicates increasing daily temperature range. Sites are ordered according to mean growth per site.


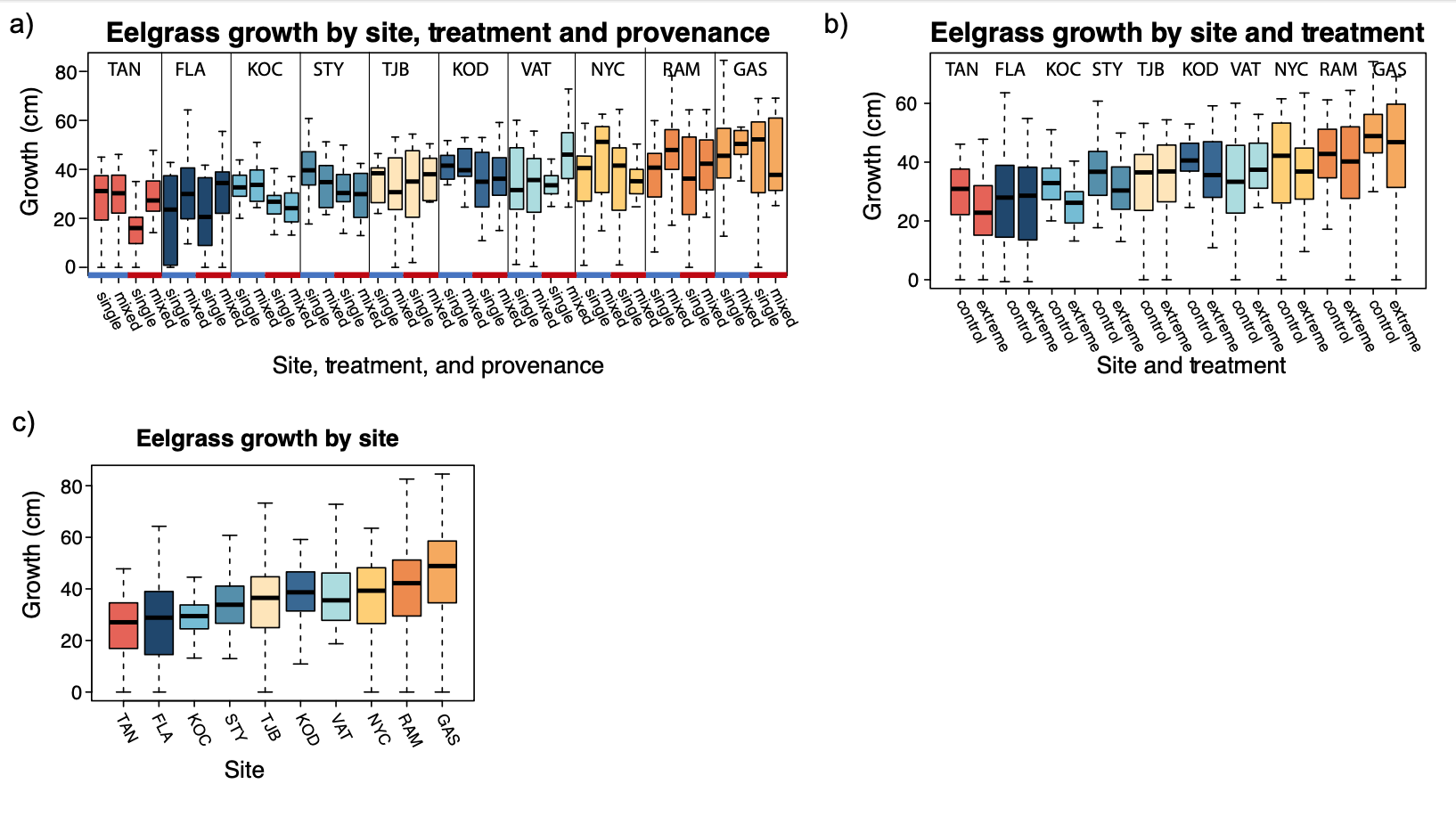


**Supplementary Figure 11.** The common garden experiment had a mean temperature difference of 3.7°C between the control and the extreme event treatment. The temperature difference was lower during the night, compared to during the day. There was little difference among the different tanks, and a clear difference between control (receiving surface water) and the warming and freshening treatment at all timepoints. Mean (solid line) and standard deviation (shading) of the 23 HOBO temperature loggers distributed over the five control tanks receiving surface water from Tjärnö bay (blue) and the five extreme treatment tanks with an elevated temperature (red). The HOBO loggers were calibrated by putting all loggers together in one water tank inside a thermoconstant room for several hours, and then calculating and applying the offset of each logger versus all others.


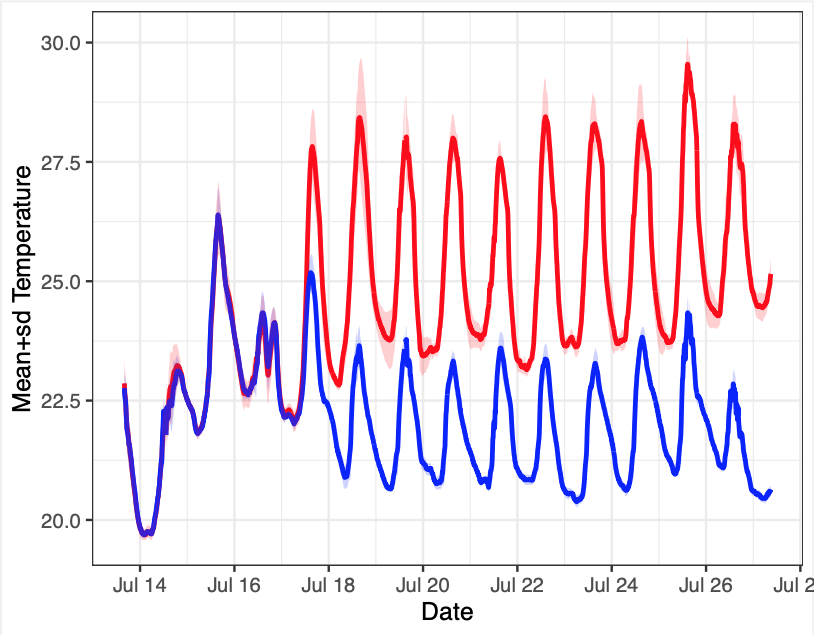


**Supplementary Figure 12.** The common garden experiment had a mean salinity difference of *ca.* 4 psu between the control and the extreme event treatment. The median of the control treatment was 23.5 ± 0.82 psu over the duration of the experiment. The treatment was freshened by ca 4 psu and had a median of 19.34 ±1.89 psu. While the median differed among experimental units in the treatment and ranged between 18.65 ± 1.55 to 20.62 ±1.47, the measures overlapped. Mean salinity measured in 4-8 cylinders for each of five control tanks receiving surface water from Tjärnö bay (green) and the five extreme event treatment tanks with an elevated temperature and decrease in salinity (blue). Salinity was measured every second day.


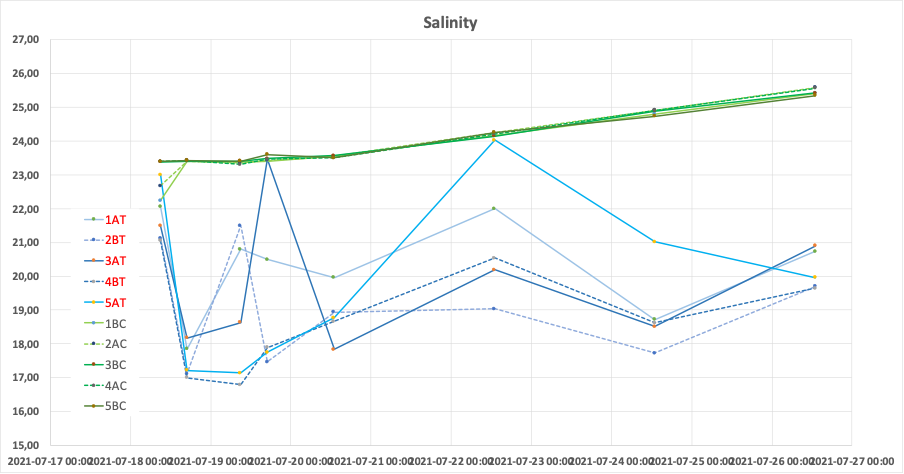


**Supplementary Tables**

**Supplementary Table 1.** Spatial and environmental information of the ten eelgrass meadows sampled in the Kosterhavet archipelago, Skagerrak, Sweden. Meadow-specific mean thermal daily maximum (Tmax), mean diurnal temperature range (TDiff), and mean daily temperature (Tmean) were recorded with Hobo loggers in each meadow for the period 5 - 29 July 2021 and 20 December 2024 - 13 February 2025. The meadows are sorted from North to South. The temperature values in cursive are the imputed values used in the RDA for Ramnekroken. As winter field work is challenging, we unfortunately only have data on winter variability for five meadows.

| **Sampling**  **site** | **Acronym** | **Lat. (°N)** | **Long. (°E)** | **Depth**  **[m]** | **Tmean**  **[°C]** | **TDiff [°C]** | **Tmax [°C]** | **organic content [%]** | **exposure** | **Tmean_winter [°C]** | **TDiff_winter [°C]** | **Tmin_winter [°C]** |
| --- | --- | --- | --- | --- | --- | --- | --- | --- | --- | --- | --- | --- |
| Styrsö | STY | 58.905591 | 11.105685 | 2.6 | 20.54 | 1.67 | 21.44 | 3.5 | EXP | - | - | - |
| Ramnekroken | RAM | 58.898535 | 11.143091 | 2.2 | *22.21* | *2.69* | *24.02* | 4.5 | SHELT | 4.53 | 1.17 | 3.89 |
| Tångeholmen | TAN | 58.895360 | 11.185677 | 1.5 | 23.03 | 2.69 | 24.44 | 4.1 | SHELT | - | - | - |
| Nycklebykilen | NYC | 58.888803 | 11.166456 | 2.0 | 22.21 | 2.06 | 23.36 | 5.3 | SHELT | 4.65 | 1.60 | 3.77 |
| Koster | KOD | 58.882538 | 10.991989 | 1.5 | 20.88 | 1.53 | 21.72 | 5.2 | EXP | - | - | - |
| Gåsholmen | GAS | 58.878628 | 11.134688 | 1.5 | 21.66 | 2.44 | 22.99 | 4.9 | SHELT | 4.97 | 1.01 | 4.23 |
| Inre  Vattenholmen | VAT | 58.877199 | 11.115649 | 2.0 | 20.80 | 1.95 | 21.93 | 2.5 | EXP | 5.25 | 0.84 | 4.82 |
| Tjärnöbo | TJB | 58.873716 | 11.191207 | 1.5 | 23.04 | 1.96 | 24.02 | 5.9 | SHELT | - | - | - |
| Flatskär | FLA | 58.856835 | 11.140161 | 2.4 | 20.76 | 1.36 | 21.42 | 5.5 | EXP | 5.39 | 0.79 | 5.02 |
| Kockholmen | KOC | 58.834280 | 11.140891 | 2.2 | 20.75 | 1.79 | 21.72 | 4.5 | EXP | - | - | - |

**Supplementary Table 2.** Sea distance among the ten eelgrass meadows collected in the Koster Sea, Swedish west coast. See Table 1 for acronyms.

|  | FLA | GAS | KOC | KOD | NYC | RAM | STY | TAN | TJB | VAT |
| --- | --- | --- | --- | --- | --- | --- | --- | --- | --- | --- |
| FLA | 0 | 3 | 2.5 | 10 | 9 | 7 | 6.5 | 7.5 | 4.5 | 3 |
| GAS | 3 | 0 | 6.5 | 9.5 | 5 | 3.5 | 4 | 6 | 5 | 1.5 |
| KOC | 2.5 | 6.5 | 0 | 10.5 | 10 | 8.5 | 8.5 | 9 | 6.5 | 5 |
| KOD | 10 | 9.5 | 10.5 | 0 | 13 | 10 | 7.5 | 12.5 | 14 | 8.5 |
| NYC | 9 | 5 | 10 | 13 | 0 | 2 | 4.5 | 2.5 | 4.5 | 4.5 |
| RAM | 7 | 3.5 | 8.5 | 10 | 2 | 0 | 3 | 3 | 5 | 3.5 |
| STY | 6.5 | 4 | 8.5 | 7.5 | 4.5 | 3 | 0 | 5.5 | 8.5 | 3.5 |
| TAN | 7.5 | 6 | 9 | 12.5 | 2.5 | 3 | 5.5 | 0 | 2.5 | 6 |
| TJB | 4.5 | 5 | 6.5 | 14 | 4.5 | 5 | 8.5 | 2.5 | 0 | 6 |
| VAT | 3 | 1.5 | 5 | 8.5 | 4.5 | 3.5 | 3.5 | 6 | 6 | 0 |

**Supplementary Table 3.** Pairwise Weir & Cockerham Fst among the ten eelgrass meadows collected in the Koster Sea, Swedish west coast. See Table 1 for acronyms.

|  | FLA | GAS | KOC | KOD | NYC | RAM | STY | TAN | TJB | VAT |
| --- | --- | --- | --- | --- | --- | --- | --- | --- | --- | --- |
| FLA | NA | 0.077 | 0.074 | 0.061 | 0.080 | 0.066 | 0.059 | 0.060 | 0.075 | 0.112 |
| GAS | 0.077 | NA | 0.060 | 0.061 | 0.028 | 0.023 | 0.039 | 0.018 | 0.032 | 0.100 |
| KOC | 0.074 | 0.060 | NA | 0.041 | 0.049 | 0.029 | 0.044 | 0.023 | 0.064 | 0.091 |
| KOD | 0.061 | 0.061 | 0.041 | NA | 0.054 | 0.042 | 0.047 | 0.036 | 0.063 | 0.100 |
| NYC | 0.080 | 0.028 | 0.049 | 0.054 | NA | 0.024 | 0.046 | 0.021 | 0.031 | 0.108 |
| RAM | 0.066 | 0.023 | 0.029 | 0.042 | 0.024 | NA | 0.035 | -0.028 | 0.034 | 0.098 |
| STY | 0.059 | 0.039 | 0.044 | 0.047 | 0.046 | 0.035 | NA | 0.026 | 0.052 | 0.070 |
| TAN | 0.060 | 0.018 | 0.023 | 0.036 | 0.021 | -0.028 | 0.026 | NA | 0.032 | 0.091 |
| TJB | 0.075 | 0.032 | 0.064 | 0.063 | 0.031 | 0.034 | 0.052 | 0.032 | NA | 0.109 |
| VAT | 0.112 | 0.100 | 0.091 | 0.100 | 0.108 | 0.098 | 0.070 | 0.091 | 0.109 | NA |

**Supplementary Table 4.** Analysis of variance (ANOVA) results for the linear model evaluating the effects of site, treatment, eelgrass provenance, and grazer diversity on growth. The model includes all main effects and their interactions (growth∼site×treatment×provenance×grazer diversity). Significant terms (p < 0.05) are highlighted in bold. Columns show the factor being tested, degrees of freedom (Df), sum of squares (Sum Sq), mean sum of squares (Mean Sq), F-statistic (F), and associated p-value (P).

| **Factor** | **Df** | **Sum Sq** | **Mean Sq** | **F** | **P** |
| --- | --- | --- | --- | --- | --- |
| site | 9 | 19203.30 | 2133.70 | 9.65 | **0.000** |
| treatment | 1 | 1660.81 | 1660.81 | 7.51 | **0.006** |
| provenance | 1 | 1607.66 | 1607.66 | 7.27 | **0.007** |
| grazer diversity | 1 | 751.55 | 751.55 | 3.40 | 0.066 |
| site:treatment | 9 | 2186.33 | 242.93 | 1.10 | 0.363 |
| site:provenance | 9 | 1903.15 | 211.46 | 0.96 | 0.476 |
| treatment:provenance | 1 | 0.11 | 0.11 | 0.00 | 0.982 |
| site:grazer diversity | 9 | 3092.59 | 343.62 | 1.55 | 0.127 |
| treatment:grazer diversity | 1 | 320.80 | 320.80 | 1.45 | 0.229 |
| provenance:grazer diversity | 1 | 157.56 | 157.56 | 0.71 | 0.399 |
| site:treatment:eprovenance | 9 | 1259.62 | 139.96 | 0.63 | 0.769 |
| site:treatment:grazer diversity | 9 | 1115.06 | 123.90 | 0.56 | 0.830 |
| site:provenance:grazer diversity | 9 | 3213.84 | 357.09 | 1.61 | 0.109 |
| treatment:provenance:grazer diversity | 1 | 0.12 | 0.12 | 0.00 | 0.981 |
| site:treatment:provenance:grazer diversity | 9 | 976.30 | 108.48 | 0.49 | 0.881 |
| Residuals | 399 | 88237.48 | 221.15 | NA | NA |

**Supplementary Table 5.** Genetic diversity for the ten eelgrass meadows with meadow code (pop), number of shoots sampled (nIndv), number of multilocus lineages (nMLL), genotypic richness (R), percentage of polymorphism corrected for sample size (Pol), the number of private alleles corrected for sample size (Pa) and inbreeding coefficient (F_IS_). Each genetic diversity estimate is reported for the clone-corrected dataset (MLL) and all samples (189). See Table 1 for acronyms.

| **pop** | **nInd** | **nMLL** | **R** | **Pol_MLL [%]** | **Pol_189 [%]** | **Pa_MLL** | **Pa_189** | **Fis_MLL** | **Fis189** |
| --- | --- | --- | --- | --- | --- | --- | --- | --- | --- |
| STY | 20 | 9 | 0.42 | 20 | 20 | 61 | 67 | -0.163 | -0.230 |
| RAM | 20 | 14 | 0.68 | 30 | 29 | 53 | 28 | -0.078 | -0.106 |
| TAN | 20 | 14 | 0.68 | 33 | 32 | 53 | 37 | -0.062 | -0.091 |
| NYC | 19 | 16 | 0.83 | 30 | 30 | 84 | 163 | -0.078 | -0.081 |
| KOD | 19 | 10 | 0.50 | 19 | 20 | 60 | 83 | -0.087 | -0.193 |
| GAS | 20 | 11 | 0.53 | 23 | 23 | 81 | 118 | -0.092 | -0.181 |
| VAT | 18 | 14 | 0.76 | 22 | 21 | 69 | 108 | -0.113 | -0.136 |
| TJB | 19 | 15 | 0.78 | 28 | 28 | 79 | 147 | -0.099 | -0.136 |
| FLA | 20 | 16 | 0.79 | 21 | 21 | 58 | 97 | -0.147 | -0.172 |
| KOC | 14 | 5 | 0.31 | 14 | 15 | 0 | 2 | -0.114 | -0.377 |

**Supplementary Table 6.** Variables included in the final full model of all 189 samples, and identified as significantly associated with genetic variation using forward variable selection with RDA (redundancy analysis). The model explains 17.7% of the genetic variation. Variables were selected through an ordiR2step procedure. ** indicates a p-value between 0.001 and 0.01.

| **Variables** | ***R*^2^ _adj_** | **Cum *R*^2^ _adj_** | ***F*-value** | ***p*-value** |
| --- | --- | --- | --- | --- |
| PC1_intergenic | 0.051 | 0.051 | 11.18 | ** |
| PC2_intergenic | 0.033 | 0.084 | 7.68 | ** |
| organic content | 0.027 | 0.111 | 6.56 | ** |
| daily temperature range | 0.025 | 0.136 | 6.52 | ** |
| PCoA1 | 0.020 | 0.156 | 5.31 | ** |
| PCoA2 | 0.020 | 0.177 | 5.55 | ** |

**Supplementary Table 7.**The influence of environment, distance and genetic structure on genetic variation in all 189 eelgrass samples when decomposed with pRDA (partial redundancy analysis). The proportion of explainable variance represents the total constrained variation explained by the full model. When basing the analysis on all individuals, more than 90% of the explainable variance is accounted for by environment, distance and genetic structure, with only a small proportion of confounding effects.

| **Partial RDA models** | ***R*^2^ _adj_** | ***p* (>*F*)** | **Proportion of explainable Variance** |
| --- | --- | --- | --- |
| Full model:  *F* ~ *env*. + *dist*. + *struct*. | 0.177 | 0.001 *** | 100% |
| Pure distance: *F* ~ *dist*. \| (*env*. + *struct*.) | 0.040 | 0.001 *** | 22.8% |
| Pure genetic structure: *F* ~ *struct*. \| (*env*. + *dist*.) | 0.077 | 0.001 *** | 43.8% |
| Pure environment: *F* ~ *env*. \| (*dist*. + *struct*.) | 0.048 | 0.001 *** | 27.3% |

**Supplementary Table 8.** List of the 22 identified putative adaptive loci identified in the clone-corrected dataset with the annotation of close-by genes.

| **outliers - dataset 124 indv - 111 MLL - (81 bp) position** | | | | | | | | |
| --- | --- | --- | --- | --- | --- | --- | --- | --- |
| # | outlier_ID | position | scaffold length | sequence ID | genes nearby | product | distance of SNP to annotated sequence | protein_id |
| 1 | Chr05_26498677 | 781596 to 781676 | 1035533 | LFYR01000864.1 | 779452..782236 | putative Auxin response factor | inside | KMZ59030.1 |
|  |  |  |  |  | 782455..783644 | hypothetical protein | 779 | KMZ59031.1 |
| 2 | Chr01_17917165 | 57461 to 57541 | 95694 | LFYR01001394.1 | 54485..55094 | hypothetical protein | 2367 | KMZ62210.1 |
|  |  |  |  |  | 93611..93710 | assembly_gap | - | NA |
| 3 | Chr02_2273959 | 2515 to 2595 | 14604 | LFYR01002162.1 | 3363..3844 | hypothetical protein | 768 | KMZ56536.1 |
|  |  |  |  | - | - | - | - | - |
| 4 | Chr06_28113541 | 558877 to 558957 | 638435 | LFYR01002101.1 | 553720..556032 | hypothetical protein | 2845 | KMZ57243.1 |
|  |  |  |  |  | 561582..562655 | myb domain protein 15 | 2625 | KMZ57244.1 |
| 5 | Chr01_17848416 | 46939 to 47019 | 88273 | LFYR01001365.1 | 37445..37544 | assembly_gap | 9395 | NA |
|  |  |  |  |  | 49827..50538 | hypothetical protein | 2808 | KMZ62406.1 |
| 6 | Chr01_18516104 | 32184 to 32264 | 56346 | LFYR01001654.1 | 30212..30463 | assembly_gap | - | NA |
|  |  |  |  |  | 36662..36781 | assembly_gap | - | NA |
| 7 | Chr01_1819599 | 116401 to 116481 | 179369 | LFYR01001020.1 | 113403..115725 | NHL repeat-containing protein | 676 | KMZ65673.1 |
|  |  |  |  |  | 116035..117583 | hypothetical protein | inside | KMZ65674.1 |
| 8 | Chr04_18271749 | 88542 to 88622 | 115934 | LFYR01001287.1 | 75700..75799 | assembly_gap | - | NA |
|  |  |  |  |  | 91185..91284 | assembly_gap | - | NA |
| 9 | Chr01_25714157 | 40254 to 40334 | 99817 | LFYR01001360.1 | 26170..27474 | Acyl-CoA oxidase | 12780 | KMZ62428.1 |
|  |  |  |  |  | 71345..82608 | assembly_gap | - | NA |
| 10 | Chr01_18329242 | 144182 to 144262 | 160926 | LFYR01001085.1 | 120026..120910 | Thaumatin-like protein 1 | 23272 | KMZ64925.1 |
|  |  |  |  |  | 151152..151251 | assembly_gap | - | NA |
| 11 | Chr01_7002036 | 423331 to 423411 | 470003 | LFYR01000235.1 | 418284..423656 | putative Vesicle-associated membrane protein | inside | KMZ74900.1 |
|  |  |  |  |  | 425723..426706 | Pectinesterase | 2312 | KMZ74901.1 |
| 12 | Chr01_15296227 | 59425 to 59505 | 185727 | LFYR01001137.1 | 41892..41991 | assembly_gap | - | NA |
|  |  |  |  |  | 67710..69728 | hypothetical protein | 8205 | KMZ64510.1 |
| 13 | Chr05_12985733 | 48709 to 48789 | 362545 | LFYR01000701.1 | 27583..40186 | MACPF domain-containing protein | 8523 | KMZ70887.1 |
|  |  |  |  |  | 45175..49192 | hypothetical protein | inside | KMZ70888.1 |
| 14 | Chr01_17646476 | 205078 to 205158 | 226709 | LFYR01000923.1 | 183810..184019 | assembly_gap | - | NA |
|  |  |  |  |  | 220159..222130 | hypothetical protein | 15001 | NA |
| 15 | Chr02_12453225 | 146240 to 146318 | 157238 | LFYR01001153.1 | 145855..145954 | assembly_gap | - | NA |
|  |  |  |  |  | 154800..154899 | assembly_gap | - | NA |
| 16 | Chr05_11602773 | 103435 to 103515 | 497251 | LFYR01000364.1 | 97931..98051 | assembly_gap | - | NA |
|  |  |  |  |  | 110430..110529 | assembly_gap | - | NA |
| 17 | Chr05_57138 | 216306 to 216386 | 802020 | LFYR01001390.1 | 194698..194854 | assembly_gap | - | NA |
|  |  |  |  |  | 223191..225078 | Vesicle-associated protein 1-1 | 6805 | KMZ62227.1 |
| 18 | Chr04_9916058 | 19271 to 19351 | 204715 | LFYR01000964.1 | 4363..5158 | assembly_gap | - | NA |
|  |  |  |  |  | 20795..21356 | putative L-asparaginase | 1444 | KMZ66575.1 |
| 19 | Chr06_28836475 | 112527 to 112607 | 350334 | LFYR01000664.1 | 106428..119431 | Phosphoribosylglycinamide formyltransferase 2 | inside | KMZ71693.1 |
|  |  |  |  |  | 122003..12264 | hypothetical protein | 9396 | KMZ71694.1 |
| 20 | Chr01_18043040 | 67152 to 67232 | 71512 | LFYR01001540.1 | 65209..65308 | assembly_gap | - | NA |
|  |  |  |  |  | 69940..70039 | assembly_gap | - | NA |
| 21 | Chr04_11506677 | 244998 to 245078 | 732666 | LFYR01001699.1 | 239149..240796 | Protein WALLS ARE THIN 1 | 4202 | KMZ59902.1 |
|  |  |  |  |  | 266120..266360 | assembly_gap | - | NA |
| 22 | Chr02_15148581 | 446662 to 446742 | 603038 | LFYR01002215.1 | 443129..444004 | hypothetical protein | 2658 | KMZ56231.1 |
|  |  |  |  |  | 448571..448792 | assembly_gap | - | NA |

**Supplementary Table 9.** List of the 11 identified putative adaptive loci identified in the dataset including all 189 samples with the annotation of close-by genes.

| **outliers - dataset 189 - (81 bp) position** | | | | | | | | |
| --- | --- | --- | --- | --- | --- | --- | --- | --- |
| # | outlier_ID | position | scaffold length | sequence ID | genes nearby | product | distance of SNP to annotated sequence | protein_id |
| 1 | Chr06_28113541 | 558877 to 558957 | 638435 | [LFYR01002101.1](https://www.ncbi.nlm.nih.gov/nuccore/LFYR01002101) | 553720..556032 | hypothetical protein | 2845 | [KMZ57243.1](https://www.ncbi.nlm.nih.gov/protein/901793437) |
|  |  |  |  |  | 561582..562655 | **myb domain protein 15** | 2625 | KMZ57244.1 |
| 2 | Chr01_18043040 | 67152 to 67232 | 71512 | LFYR01001540.1 | 65209..65308 | assembly_gap | - | NA |
|  |  |  |  |  | 69940..70039 | assembly_gap | - | NA |
| 3 | Chr05_19337062 | 821099 to 821179 | 835807 | LFYR01001470.1 | 792060..819561 | assembly_gap | - | NA |
|  |  |  |  |  | 826646..827230 | assembly_gap | - | NA |
| 4 | Chr04_2146255 | 357820 to 357900 | 522578 | LFYR01000158.1 | 345622..348672 | Pre-mRNA-processing protein | 9148 | KMZ75553.1 |
|  |  |  |  |  | 357573..364362 | Protein BONZAI 1 | inside | KMZ75554.1 |
| 5 | Chr05_6978637 | 325097 to 325177 | 413393 | LFYR01000612.1 | 323723..324054 | assembly_gap | - | NA |
|  |  |  |  |  | 325681..325940 | assembly_gap | - | NA |
| 6 | Chr05_20427352 | 607253 to 607333 | 878598 | LFYR01001125.1 | 593809..594296 | hypothetical protein | 12957 | KMZ64619.1 |
|  |  |  |  |  | 608371..611067 | Adenosylmethionine decarboxylase | 1038 | KMZ64620.1 |
| 7 | Chr05_24279728 | 373122 to 373202 | 1562992 | LFYR01001430.1 | 363080..371053 | GTPase HflX | 2069 | KMZ61753.1 |
|  |  |  |  |  | 371517..373337 | CASP-like protein | inside | KMZ61754.1 |
| 8 | Chr06_21768194 | 91051 to 91131 | 199296 | LFYR01001024.1 | 85724..113839 | Signal peptide peptidase SppA, 67K type | inside | KMZ65639.1 |
|  |  |  |  |  | 115460..119350 | Prefoldin subunit | 24329 | KMZ65640.1 |
| 9 | Chr02_31020419 | 81367 to 81447 | 180271 | LFYR01001262.1 | 63848..74854 | assembly_gap | - | NA |
|  |  |  |  |  | 82021..82120 | assembly_gap | - | NA |
| 10 | Chr06_16144718 | 51310 to 51390 | 317578 | LFYR01000784.1 | 35046..39070 | assembly_gap | - | NA |
|  |  |  |  |  | 71686..71785 | assembly_gap | - | NA |
| 11 | Chr02_31212552 | 566889 to 566969 | 802020 | LFYR01001390.1 | 552913..553012 | assembly_gap | - | NA |
|  |  |  |  |  | 584289..584560 | hypothetical protein | 17320 | [KMZ62261.1](https://www.ncbi.nlm.nih.gov/protein/901802528) |
